# Transcriptome Analysis of *Chloris virgata*, Which Shows the Fastest Germination and Growth in the Major Mongolian Grassland Plant

**DOI:** 10.3389/fpls.2021.684987

**Published:** 2021-06-28

**Authors:** Byambajav Bolortuya, Shintaro Kawabata, Ayumi Yamagami, Bekh-Ochir Davaapurev, Fuminori Takahashi, Komaki Inoue, Asaka Kanatani, Keiichi Mochida, Minoru Kumazawa, Kentaro Ifuku, Sodnomdarjaa Jigjidsuren, Tugsjargal Battogtokh, Gombosuren Udval, Kazuo Shinozaki, Tadao Asami, Javzan Batkhuu, Takeshi Nakano

**Affiliations:** ^1^Graduate School of Biostudies, Kyoto University, Kyoto, Japan; ^2^School of Engineering and Applied Sciences, National University of Mongolia, Ulaanbaatar, Mongolia; ^3^Gene Discovery Research Group, RIKEN Center for Sustainable Resource Science, Tsukuba, Japan; ^4^Bioproductivity Informatics Research Team, RIKEN Center for Sustainable Resource Science, Yokohama, Japan; ^5^Research Institute of Animal Husbandry, Mongolian University of Life Science, Ulaanbaatar, Mongolia; ^6^Department of Applied Biological Chemistry, The University of Tokyo, Tokyo, Japan

**Keywords:** plant growth, transcriptome, germination, plant hormone, regrowth, brassinosteroid, *Chloris virgata*

## Abstract

Plants in Mongolian grasslands are exposed to short, dry summers and long, cold winters. These plants should be prepared for fast germination and growth activity in response to the limited summer rainfall. The wild plant species adapted to the Mongolian grassland environment may allow us to explore useful genes, as a source of unique genetic codes for crop improvement. Here, we identified the *Chloris virgata* Dornogovi accession as the fastest germinating plant in major Mongolian grassland plants. It germinated just 5 h after treatment for germination initiation and showed rapid growth, especially in its early and young development stages. This indicates its high growth potential compared to grass crops such as rice and wheat. By assessing growth recovery after animal bite treatment (mimicked by cutting the leaves with scissors), we found that *C. virgata* could rapidly regenerate leaves after being damaged, suggesting high regeneration potential against grazing. To analyze the regulatory mechanism involved in the high growth potential of *C. virgata*, we performed RNA-seq-based transcriptome analysis and illustrated a comprehensive gene expression map of the species. Through *de novo* transcriptome assembly with the RNA-seq reads from whole organ samples of *C. virgata* at the germination stage (2 days after germination, DAG), early young development stage (8 DAG), young development stage (17 DAG), and adult development stage (28 DAG), we identified 21,589 unified transcripts (contigs) and found that 19,346 and 18,156 protein-coding transcripts were homologous to those in rice and *Arabidopsis*, respectively. The best-aligned sequences were annotated with gene ontology groups. When comparing the transcriptomes across developmental stages, we found an over-representation of genes involved in growth regulation in the early development stage in *C. virgata*. Plant development is tightly regulated by phytohormones such as brassinosteroids, gibberellic acid, abscisic acid, and strigolactones. Moreover, our transcriptome map demonstrated the expression profiles of orthologs involved in the biosynthesis of these phytohormones and their signaling networks. We discuss the possibility that *C. virgata* phytohormone signaling and biosynthesis genes regulate early germination and growth advantages. Comprehensive transcriptome information will provide a useful resource for gene discovery and facilitate a deeper understanding of the diversity of the regulatory systems that have evolved in *C. virgata* while adapting to severe environmental conditions.

## Introduction

Mongolia is a highland country with an average height of 1580 m, including steppe grassland, mountains, and desert. The Mongolian climate is cold and dry with long winters, short summers, and a wide temperature range between winter and summer. The precipitation level is extremely low and occurs mainly in summer between June and September. The main grasslands, including the steppe, mountain steppe, and forest areas, receive 200–300 mm precipitation annually (Suttie, 2006). As summer in the Mongolian grassland is very short and dry, almost all plants have prepared to germinate and grow quickly, making use of the small amount of rain and the short warm period, which starts in July. Plant growth ceases after August owing to low temperatures and frosts. These severe climatic conditions have suppressed and screened Mongolian grassland plants. Unique and high-performance plants have evolved by surviving this natural selection.

About 80% of Mongolian territory is covered by natural plants and used as natural pasture by nomadic groups, with extensive cultivation of livestock such as goats, sheep, cattle, horses, and camels. Mongolia is mainly engaged in animal husbandry; as of 2019 the number of livestock reached about 71 million, an increase of 6.4% compared to the previous year. As approximately 90% of the livestock rely on natural pastureland for their feed, extreme weather events such as droughts and ‘dzud’ (a local term for severe winter conditions that affect livestock) that causes decreasing of natural pasture have severe effects on the livestock in the nomadic pasture system. The highest annual growth of pasture plants occurs in August. However, in recent years, annual growth has decreased to 75% of the highest growth owing to drought and the degradation of grazing lands by overgrazing and exceeding the carrying capacity (Karban and Baldwin, 1997). Every season, Mongolian nomads move to convenient new areas to search for the best pasture to meet the nutritional demands of their livestock. These nomadic cultures might also be important for research on the growth mechanisms of Mongolian natural plants.

In recent years, most Mongolian grassland plants have been analyzed in the hopes of uncovering information useful to phytochemical and pharmacological research. Natural wild plants from 3160 species, 564 genera, and 128 families live in Mongolia, over a wide temperature range of four seasons (Grubov, 2008). Approximately 600 species have been identified as significant edible plants and 1100 species as medicinal plants. Furthermore, 150 species are rich vitamin resources, 200 contain essential oils, 250 contain tannins, more than 200 are used for dyeing, 231 are rich in flavonoids, 200 can be used as ingredients in traditional medicine, more than 480 are ornamental plants, 280 contain alkaloids, and 65 are used to prevent desertification (Jamyandorj et al., 2011). The Western Pacific Region of the World Health Organization have listed the most useful medicinal plants found in Mongolia (Shin, 2013).

The main Mongolian rangeland plants include 26 families and 73 genera, among which the Compositae and Gramineae families are the most abundant, both easily distributed by wind (Lapin et al., 2017). *Agropyron cristatum* is one of well eatable plant for animal husbandry in Mongolia and is also widely distributed in European and Asian grasslands. Additionally, *A. cristatum* is a possible gene donor for wheat improvement, including resistance to wheat streak mosaic virus and leaf rust disease (Sharma et al., 1984; Ochoa et al., 2015). In recent years, a comprehensive transcriptome analysis of *A. cristatum* has been performed for the investigation of functional genes (Zhou et al., 2019), and many stress-resistance genes have been identified (Zhang et al., 2015).

In this study, we screened Mongolian grassland plants for high growth ability and identified the *Chloris virgata* Dornogovi (DG) accession. To obtain a comprehensive transcriptome of *C. virgata*, RNA-seq was performed to gain insight into genetic regulation during the germination, early development, young development, and adult development stages of *C. virgata*, and the transcriptome of *C. virgata* is presented here for the first time. Complete genome sequencing has only been used to reveal a few complete genomes as the method faces several challenges, including the complexity of complete genomes, the sequencing costs, and the computational resources required; therefore, it is not suitable for non-model plants. Instead, RNA sequencing can be used for non-model plants without a reference genome, and has become the most convenient and cost-effective tool for gaining insight into transcriptome profiling and for detecting differentially expressed genes (DEGs) (Wang et al., 2009). For instance, RNA sequences for crops such as eggplants (Ramesh et al., 2016), peppers (Gordo et al., 2012), tobacco (Lei et al., 2014), corn (Lu et al., 2017), grapes (Xu et al., 2014), jute (Yang et al., 2017), and cotton (Wei et al., 2017), as well as wild plants such as *Craterostigma plantagineum* (Rodriguez et al., 2010) and *A. cristatum* (Zhang et al., 2015) have been published. However, only the complete chloroplast genome sequence of *C. virgata* is available in the National Center for Biotechnology Information (NCBI) database (Hereward et al., 2016).

In this study we establish, for the first time, a *de novo* transcriptome assembly for *C. virgata* at four different developmental stages. We present a total of 43,752,426 raw RNA-seq reads and use them for the *de novo* assembly and annotation of genes from *C. virgata* against *Arabidopsis* and rice. DEGs were identified at each of the four developmental stages and these data were used for phytohormone homolog searching. Our findings may serve as a basis for further gene discovery research on *C. virgata.*

## Materials and Methods

### Plant Materials and Growth Conditions

Seeds of the *C. virgata* DG accession were harvested from the native grassland of DG Province in Mongolia and stored at 25 ± 2°C. Rice (*Oryza sativa* L. cv. Nipponbare), wheat (*Triticum aestivum* L. cv. Norin No. 61), and *Arabidopsis thaliana* ecotype Columbia (hereafter *Arabidopsis*) were used as control plants. The 9 de-husked seeds per each plant were sterilized in 5% sodium hypochlorite and 0.07% Tween-20 for 20 min and rinsed three times with sterile water for 10 min. After sterilization, the seeds were sown on 1/2 Murashige and Skoog basal (MS, Duchefa-Biochemie, Haarlem, Netherlands) plates containing 1.5% sucrose and 0.9% phytoagar (Duchefa-Biochemie, Haarlem, Netherlands). After 2 days of incubation at 4°C in the dark, the seeds were incubated at 22°C with a 16-h light/8-h dark photoperiod for germination analysis. For the observation of plants grown in soil, the seedlings were transplanted into soil 7 days after germination (DAG) and grown at 22°C with a 16-h light/8-h dark cycle. These experiments was performed with three biological replicates.

### Regrowth Potential Analysis

The leaf regrowth potential after animal bite was tested by cutting the leaves with scissors to mimic cutting by animal teeth. After 2 weeks of growth in soil, the plants were cut back to a height of 4 cm from the soil surface using scissors. Cutting was performed every 2 weeks for 12 weeks (six cutting events).

### RNA Extraction

Total RNA was extracted from plants grown at four different developmental stages: the germination, early young development, young development, and adult development stages. *C. virgata* were grown in 1/2 MS medium supplemented with 1.5% sucrose and 0.9% phytoagar at 22°C under white light (a 6-h light/8-h dark cycle was used for long-day conditions). The *C. virgata* grown on 1/2 MS were harvested 2 days (germination stage) and 8 days (early young development stage) after germination. *C. virgata* grown in soil were harvested 10 days (young development stage) and 21 days (adult development stage) after transplantation of 7-day-old plants from 1/2 MS to the soil. For the whole-plant samples, only the shoots were collected and the roots cut away. Total RNA was extracted from the plant samples using the RNeasy Plant Mini kit (Qiagen).

### cDNA Synthesis and RNA-Seq Analysis Using an Illumina Sequencing System

To create a cDNA sequencing library, mRNAs with poly (A) tails were isolated from the total RNA using the NEBNext^®^ Poly (A) mRNA Magnetic Isolation Module (NEB E7490) and NEBNext^®^ Ultra RNA Library Prep Kit for Illumina^®^ (E7530). The first strand of cDNA was synthesized using random hexamer primers. The cDNA fragment was amplified using the 1.8 × Agencourt AMPure XP Beads linker and NEBNext Adaptor for Illumina. The Illumina NovaSeq^TM^ sequencing system was used for sequencing. The raw reads dataset was submitted to the DNA Data Bank of Japan Sequence Read Archive (DDBJ SRA) under accession number DRA011714.

### *De novo* Transcriptome Assembly and Sequence Clustering

The RNA-seq raw reads were trimmed using Trimmomatic (v0.39) (Bolger et al., 2014) with the following parameter settings: TLEADING:30 TRAILING:30 SLIDINGWINDOW:4:15 MINLEN:60. The trimmed reads were assembled using Trinity (v2.8.5) (Grabherr et al., 2011) with the parameters ‘seqType fq –SS_lib_type RF.’ Trinity-based contigs were further assembled using the PCAP assembler (Huang et al., 2003). The PCAP-based contigs were clustered using CD-HIT-EST (v.4.8.1) (Fu et al., 2012) with the identity parameter set to ≥80. Using the longest sequences in each of the CD-HIT-EST clusters as unique transcripts, protein-coding sequence regions were predicted using TransDecoder (v.5.5.0)^1^ with the results of a sequence similarity search and domain search against the UniProt and Pfam-A databases, conducted using BLASTx search (NCBI BLAST v2.8.1) and the hmmscan program in the HMMER package (v.3.2.1) (Johnson et al., 2010), respectively. Unique transcripts containing protein-coding sequences were used for downstream analyses as mapping references.

### Functional Annotation and Gene Ontology Enrichment Analysis

To predict the gene function of the deduced protein sequences from the reference transcripts of *C. virgata*, their closest homologs in rice and *Arabidopsis* were searched for in the TAIR10 and IRGSP1.0 (Ensemble plants release-44) using the BLASTp program with a cutoff *E*-value < 10^–5^, and swissprot (v5) database using the CloudBlast function of OmixBox software (v.1.1.164) with a cutoff *E*-value < 10^–5^. The reference *C. virgata* transcripts were annotated with gene ontology (GO) terms using OmicsBox, and gene-set enrichment analysis was conducted using the GO terms of DEGs, based on the Fisher’s exact test function (false discovery rate, FDR < 0.03) in OmicsBox.

### cDNA Synthesis and RNA-seq Analysis by Ion Proton Sequencing

The Thermo Fisher Ion Proton^TM^ sequencing system was used for RNA-seq analysis to identify DEGs in *C. virgata.* A Dynabeads mRNA Purification Kit and Ion Total RNA-Seq Kit v2 (Thermo Fisher Scientific) were used for the purification of mRNA from the total RNA samples and the construction of a cDNA library, respectively. The quality of the total RNA, mRNA, and cDNA libraries were analyzed with an Agilent Tape Station (Agilent Technologies). The cDNA libraries were pooled for emulsion PCR using an Ion PI Hi-Q Chef Kit (Thermo Fisher Scientific). The enriched samples were loaded onto an Ion PI chip (v3) using Ion Chef and sequenced using an Ion Proton instrument. The raw reads dataset was submitted to the DDBJ SRAunder accession number DRA011714.

### Expression Profiling and Differentially Expressed Genes

The trimmed RNA-seq reads from the ion proton sequencing were mapped onto the reference transcript dataset using TMAP (v. 3.0.1)^2^. The expression level of each transcript was quantified based on read count data computed by the featureCounts program (Liao et al., 2014) and normalized to reads per million mapped reads (RPM). Differentially expressed transcripts between pairwise combinations of samples were identified using the Pairwise Differential Expression Analysis function based on edgeR (Robinson et al., 2009) with the read count data output from the Create Count Table function based on HT-seq (Anders et al., 2015) performed with OmixBox.

### Phytohormone Similarity Sequence Searching and Alignment

Phytohormone similarity was assessed between *C. virgata* and the *A. thaliana* (accession number: GCF_000001735.4) and *O. sativa* (accession number: GCF_000005425.2) protein sequences downloaded from the NCBI. Similarity searches were conducted using BLASTx 2.9.0 + (*E-*value < 1e-5). Highly similar sequences were clustered using CD-HIT 4.8.1, with 99% identity, and each cluster was used for read alignment. Multiple sequence alignment was performed using MAFFT 7.470 (Katoh and Standley, 2013) with the <–auto> commands. All columns with gaps in more than 50% of the sequences were removed using TrimAL 1.4 and the maximum likelihood phylogenetic tree was created using IQ-Tree 1.6.12 (Nguyen et al., 2015) with 1000 bootstrap replicates (Hoang et al., 2018) and Model Finder (Kalyaanamoorthy et al., 2017).

### Phylogenetic Analysis

We downloaded the protein sets of the whole chloroplast genomes of 17 species, including *C. virgata*, from the NCBI RefSeq and GenBank databases. Single-copy orthologs of all genomes were extracted using OrthoFinder 2.4.0 (Emms and Kelly, 2019) and all single-copy orthologs were aligned using MAFFT 7 (Katoh and Standley, 2013) <–auto> and TrimAL <automated>. The maximum likelihood tree was inferred by IQ-Tree 1.6 software (Nguyen et al., 2015) with 1000 bootstrap replicates (Hoang et al., 2018) and Model Finder (Kalyaanamoorthy et al., 2017).

## Results

### *Chloris virgata* Showed the Fastest Growth Phenotype of the Major Mongolian Grassland Plants

To screen for plants harboring fast growth potential, 40 species of major Mongolian grasslands plants were collected from the northern forest zone, middle grass zone, and southern Gobi desert zone of the Mongolian territory. Additionally, the agricultural crops, rice and wheat, and the representative molecular biology experimental plant, *Arabidopsis*, were used as controls during seed screening. The seeds were sown on 1/2 MS medium containing 0.9% agarose and 1.5% sucrose and incubated at 4°C for 2 days for vernalization. The seed germination treatment was started by moving the seeds to 22°C under 16-h light/8-h dark conditions. The first appearance of shoot were defined as a starting of germination and growth phenotypes were observed using a stereoscopic microscope. The germination of wheat and rice was observed 1 and 2 days, respectively, after the start of germination treatment. *Arabidopsis* germinated after 2 days. Of the Mongolian grassland plants, *Agropyron cristatum* and *Medicago* spp. that were used as feed for livestock germinated in 1 or 2 days. In contrast with these well-known plants, the *C. virgata* DG accession was observed to germinate just 5 h after the start of germination treatment and was identified as the fastest germinating plant of the 43 screened seeds (Table 1). *C. virgata* is an annual monocot belonging to the Poaceae family. The English common name of *C. virgata* is “feather finger grass” and it is also called ‘bulgan suul’ in Mongolia, which means “tail of sable.”

**TABLE 1:** *Chloris virgata* showed the fastest germination of the major Mongolian grassland plants.

Plant name	Germination time
*Chloris virgata*	5 hrs
*Agropyron cristatum*	1 day
*Medicago sativa*	1 day
*Rheum undulatum*	1 day
Wheat (*Triticum aestivum*)	1 day
*Arabidopsis mongolica*	2 days
*Arabidopsis thaliana*	2 days
*Astragalus tibetanus*	2 days
*Medicago falcata*	2 days
*Medicago varia*	2 days
*Melilotus* sp.	2 days
*Melilotus dentatus*	2 days
Rice (*Oryza sativa*)	2 days
*Setaria viridis*	2 days
*Zygophyllum xanthoxylon*	2 days
*Caragana microphylla*	3 days
*Astragalus mongolica*	4 days
*Astragalus propinquus*	4 days
*Polygonum divaricatum*	4 days
*Polygala tenuifolia*	4 days
*Schizonepeta multifida*	4 days
*Silene repens*	4 days
*Veronica incana*	4 days
*Agrostis mongolica*	5 days
*Astragalus monophyllus*	6 days
*Lathyrus prantense*	6 days
*Onobrychis sibirica*	6 days
*Stipa krylovii*	6 days
*Allium senescens*	7 days
*Gallium verum*	7 days

The complete chloroplast genomes of 14 common species and two *Chloris* species provided an analysis of the maximum likelihood relationships between *C. virgata* and the other 16 species. All chloroplast genomes were downloaded from NCBI RefSeq and the GenBank database using the chloroplast genome of *C. virgata*. The dicots *A. thaliana* and *Medicago sativa* were used as outgroups. Phylogenetic analysis of the 14 common species and two *Chloris* species revealed that the *Chloris* species are most closely related to *Zoysia* grasses (Supplementary Figure 2).

**FIGURE 1:**
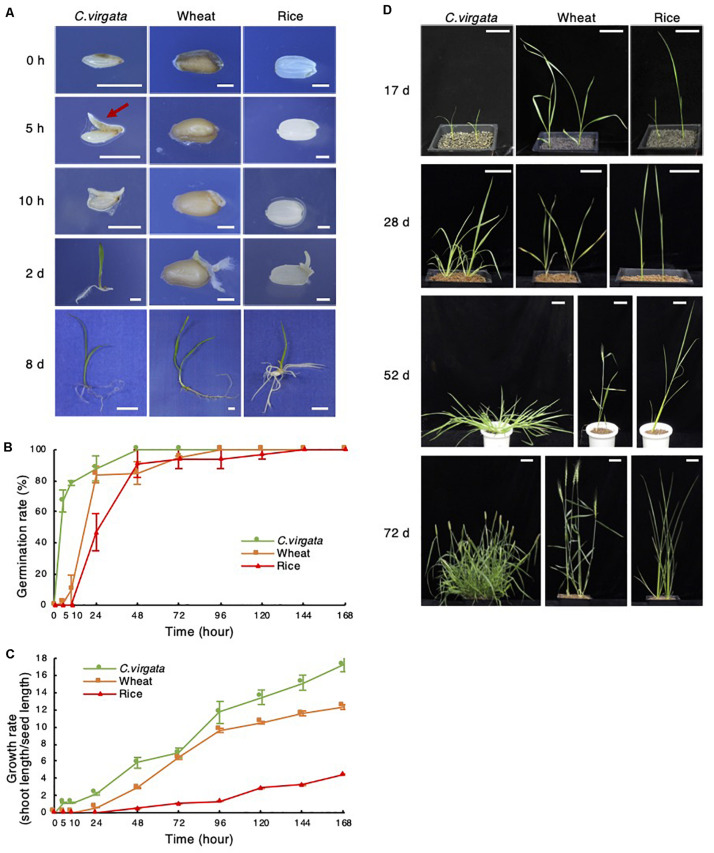
*Chloris virgata* possessed faster germination ability than wheat and rice. **(A)** Shoot and root formation in the germination stage of *C. virgata*, wheat, and rice. Each plant was germinated on 1/2 MS medium for 0, 5, 10 h, 2 days, or 10 days. Scale bars: 2 mm (0 and 10 h), 5 mm (2 days), and 1 cm (8 days). Seed germination rate **(B)** and growth rate **(C)** of *C. virgata*, wheat, and rice from 0 to 7 days after germination. Totally 27 plants were measured with three independent replicates. **(D)** Shoot and branch development from the early to young development stages. Each plant was germinated and grown on soil for 17, 28, 52, or 72 days. Scale bars: 5 cm.

Only 5 h after the start of germination treatment, a tiny shoot appeared at the bottom of the *C. virgata* seed, whereas no shoots or roots emerged from the wheat or rice seeds (Figure 1A). For a detailed analysis of germination potential, germination efficiency was analyzed to measure the rate of shoot or root formation in each period after the start of germination treatment (Figure 1B). In the 5 h stage, the germination rate of *C. virgata* seeds was approximately 60%, which is significantly faster than that of the other plants (Figure 1B). In contrast, wheat showed a 10% germination rate at 10 h, and rice showed about a 30% germination rate at 24 h (Figure 1B). Growth potential is analyzed not only using germination speed, but also the shoot formation of each plant. Consequently, the shoot phenotypes and lengths of the *C. virgata*, wheat, and rice shoots were analyzed. Shoots length per seed length was measured for 7 days after the start of germination (Figure 1C). The *C. virgata* shoots elongated to 200 and 380% of seed length within 24 and 48 h of starting the germination treatment, respectively. The wheat and rice seeds only started to form shoots 24 and 48 h after the start of germination treatment, respectively. *C. virgata* continued to show faster shoot growth than the wheat or rice for 7 DAG (Figure 1C).

To observe growth potential in later stages, the leaf and stem development of *C. virgata*, wheat, and rice were observed from the early young to adult development stages. Each plant seed was germinated on 1/2 MS medium for 7 days under 16-h light/8-h dark conditions, and the early young development plants were transplanted to soil and grown at 22°C under 16-h light/8-h dark conditions (Figure 1D). From 17 to 28 DAG, increased branch formation was observed in *C. virgata*. The active branch formation of *C. virgata* continued until day 52, and the branch number was higher than those of wheat or rice. *C. virgata* branch formation was observed until the seed formation stage at 72 days. The final branch number of *C. virgata* was found to be over 20 per plant, which is substantially more than the 3–5 branch formations in wheat and rice (Figure 1D).

### *Chloris virgata* Showed a Strong Regrowth Phenotype

**FIGURE 2:**
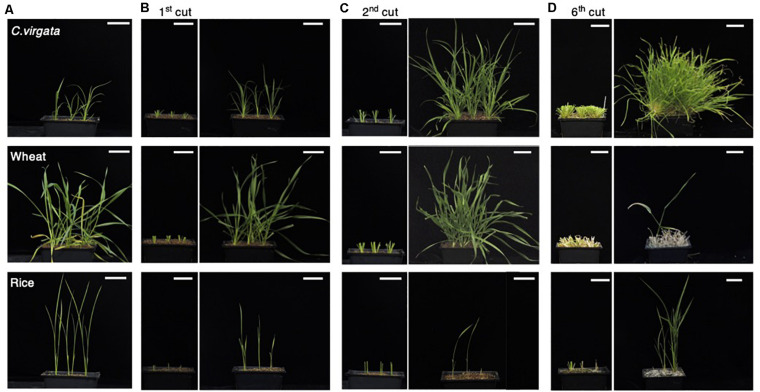
*Chloris virgata* possessed higher regeneration ability after shoot cutting than wheat and rice. **(A)** *C. virgata*, rice, and wheat were grown on soil for 2 weeks. **(B)** The shoots of the two-week-old plants were cut and removed (left). The shoots were regrown for 2 weeks after the first cutting treatment (right). **(C)** Regrown shoots after the first cutting treatment were recut a second time (left). The plants were regrown for 2 weeks after the second cutting treatment (right). **(D)** Remaining shoots after the sixth cutting treatment (left) and the regrown shoots after 2 weeks (right). Scale bars: 5 cm.

As nomadism is a traditional Mongolian way of life, many plant species growing on Mongolian grasslands are exposed to grazing. The nomadic culture in Mongolia dictates that *C. virgata* should preferably be eaten by sheep, but *C. virgata* showed regrowth after animal bite by Mongolian livestock. To investigate the regrowth potential of this species, the leaves and stems of *C. virgata*, wheat, and rice were cut to a height of 4 cm from the soil surface using scissors to mimick animal bite. The regrowth potential of the plants was observed for 2 weeks between cuttings. Initially, the 7-day-old *C. virgata*, wheat, and rice seedlings were transplanted to soil and grown for 2 weeks (Figure 2A). The first cutting treatments were then performed (Figure 2B, left). After the first cutting treatment, the leaves and stems of all the plants were regenerated and regrown over the next 2 weeks (Figure 2B, right). Then, the second cutting treatment was performed on the recovered plants (Figure 2C, left). After the second cutting, *C. virgata* and wheat showed better regrowth phenotypes, with higher branch numbers and shoot heights than the regrowth phenotypes after the first cutting treatment. The growth volume of rice after the first and second cutting treatments was less than those of *C. virgata* and wheat (Figure 2C, right). During the third to fifth regrowth period, *C. virgata* continued to regrow with the same shoot number and height as after the second cutting, but the regrowth shoot number and height of the wheat gradually decreased. Rice growth continued, but with low shoot numbers and heights (Supplementary Figures 1A–C, left). After the sixth cutting, the shoots of *C. virgata* still fully regrew, without any visible growth depression. In contrast, wheat regrowth declined after the sixth cutting, and the number and height of the shoots drastically decreased (Figure 2D, right). These results suggest that *C. virgata* has high regrowth ability against grazing, compared to the common commercial crops, wheat and rice.

### *De novo* Transcriptome Assembly and Functional Annotation of *Chloris virgata*

To analyze the molecular mechanisms of *C. virgata* growth activities, the total RNA extracted from samples of four independent growth stages were pooled and then subjected to Illumina RNA sequencing and *de novo* transcriptome assembly. A total of 43,752,426 raw sequence reads were generated from the *C. virgata* cDNA, and 41,764,866 filtered reads (Table 2). The quality-checked reads were assembled *de novo* using the Trinity program (Grabherr et al., 2011), resulting in 121,418 contigs with an N50 value of 1933 bp. These were assembled into 31,955 super-contigs with the PCAP program and clustered using the CD-HIT-EST program, resulting in 28,173 transcript clusters. The longest transcripts of the clusters were then subjected to open reading frames, resulting in 21,589 protein-coding transcripts, which were used in our downstream analyses as the reference transcript dataset. In the reference transcripts, 19,346 and 18,156 transcripts were homologous to protein-coding genes in *O. sativa* and *Arabidopsis*, respectively (Table 2).

**TABLE 2:** Summary of transcriptome sequence assembly from *Chloris virgata* after Illumine sequencing.

	No. sequences	N50
Number of raw sequence reads	43,752,426	
Number of raw sequence reads trimed	41,764,866	
Number of contigs (Trinity)	121,418	1,933
Number of super-contigs (pcap)	31,955	2,260
Number of clusters (cd-hit-est)	28,173	2,237
Number of contigs with predicted ORFs (TransDecoder)	21,589	
Number of ORFs homologous to protein coding genes in *O. sativa*	19,346	
Number of ORFs homologous to protein coding genes in *A. thaliana*	18,156	

### Functional Annotation of Expressed Genes in *Chloris virgata*

**FIGURE 3:**
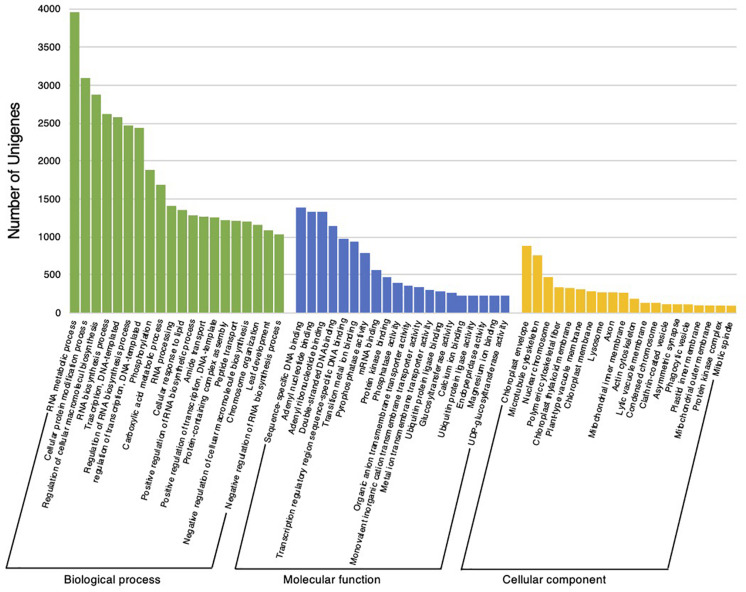
Functional classification of *Chloris virgata* assembled unigenes. Gene ontology terms for all unigenes sequences were assigned using a BLASTx search. The results were summarized into the following categories: biological process, molecular function, and cellular component.

The reference transcripts derived from the *C. virgata* transcriptome assembly were assessed using a homology search and GO annotation. The homology search was conducted against the NCBI non-redundant protein database for the reference transcripts, using the BLASTx program, and the highest hits were linked to the reference transcripts. This resulted in an aligned sequence homology of 75.5% with the NCBI-nr database. The data were further subjected to GO-based functional classification in OmicsBox software, to illustrate the annotated gene profiles in 60 GO categories related to biological process (20), molecular function (20), and cellular component (20) (Figure 3). Among the biological process categories, RNA metabolic process, cellular protein modification process, and regulation of cellular macromolecule biosynthesis were ranked the highest. Within the molecular function category, binding functions, including sequence-specific DNA binding, adenyl nucleotide binding, and double-stranded DNA binding, were ranked the highest. In addition, in the cellular component category, the chloroplast envelope, microtubule cytoskeleton, and nuclear chromosome were ranked the highest (Figure 3).

### Gene Expression Profiles in the Juvenile Development Stage of *Chloris virgata*

**FIGURE 4:**
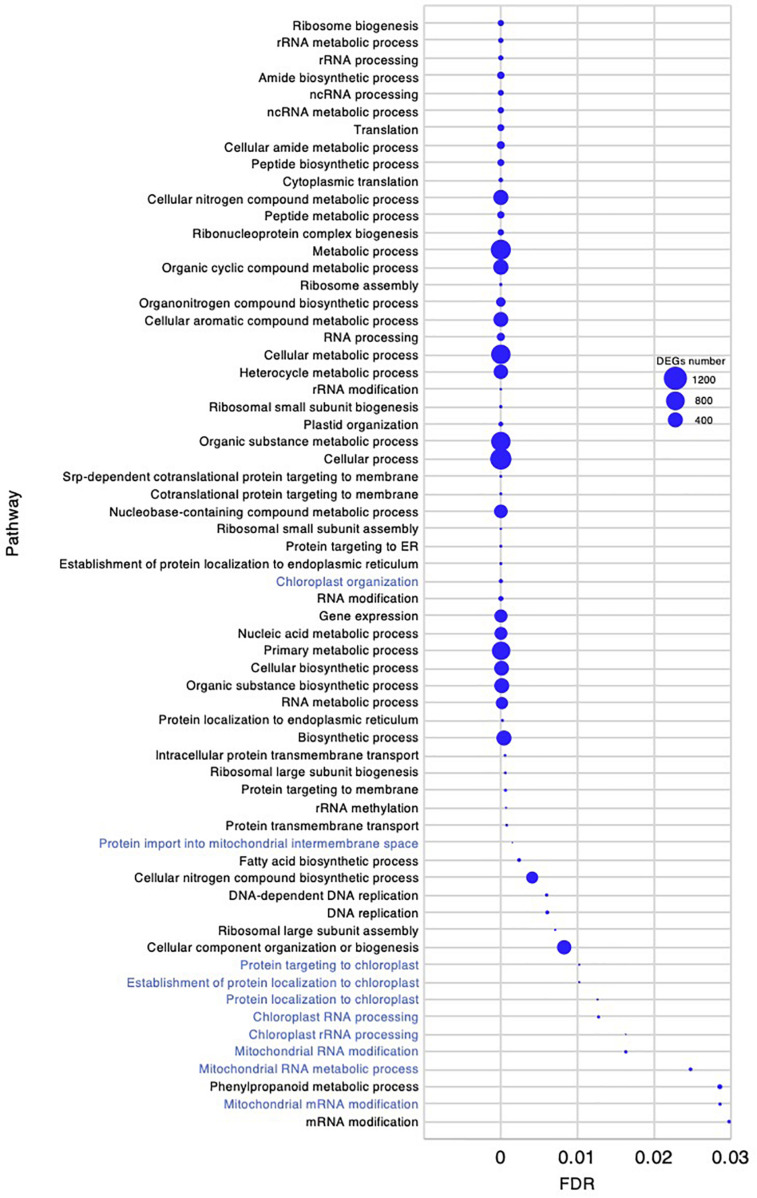
Gene ontology enrichment analysis in the germination stage of *Chloris virgata.* Gene ontology terms of differentially expressed genes (DEGs) enriched in the germination stage (2 days after germination), compared with the early young development stage (8 days after germination) of *C. virgata* grown on 1/2 MS medium. Gene ontology terms were selected from the biological process category. A false discovery rate (FDR) of 0.03 was used as the threshold. Blue circles show the number of DEGs in each Gene ontology term.

**FIGURE 5:**
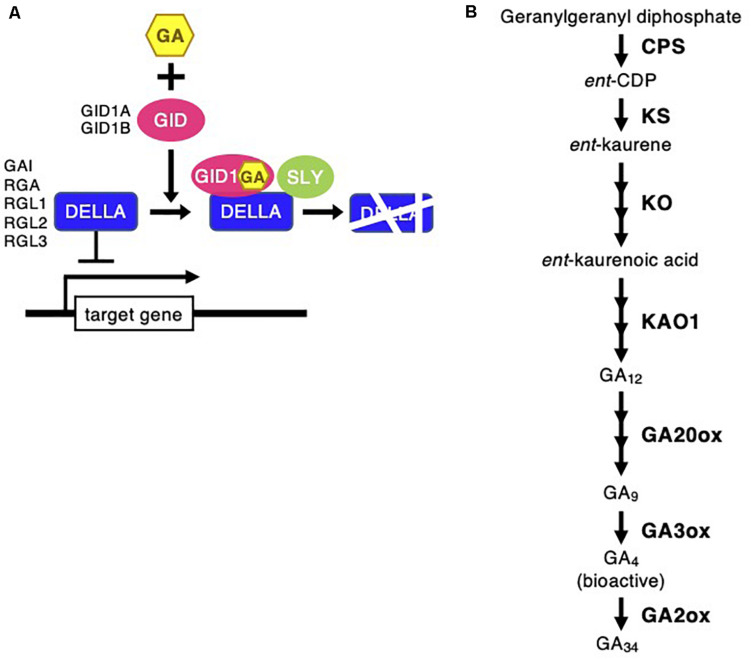
Gibberellin signaling and biosynthesis pathway following *Chloris virgata*. **(A)** The possible GA signaling pathway in *C. virgata.* GID, gibberellin-insensitive dwarf; GAI, gibberellic acid insensitive; RGA, repressor of GA; RGL, RGA-like; SLY, sleepy. **(B)** GA biosynthesis pathway in *C. virgata*. GAs are products of diterpenoid pathway and their formation from GGDP to bioactive GAs such as GA_4_. CPS, *ent-*copalyl diphosphate synthase; GGDP, geranylgeranyl diphosphate; *ent-*CDP, *ent-*copalyl diphosphate; KS, *ent-*kaurene synthase; KO, *ent-*kaurene oxidase; KAO, *ent-*kaurenoic acid oxidase. The arrows indicate each steps.

**FIGURE 6:**
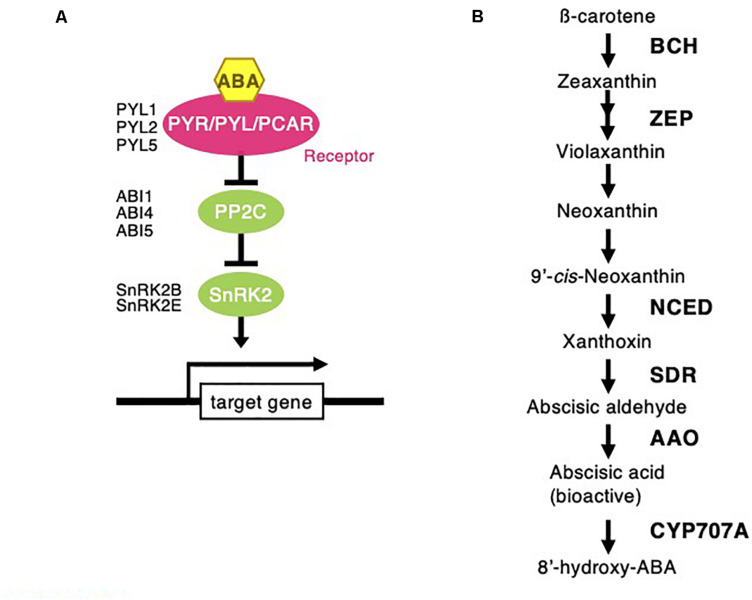
Abscisic acid signaling and biosynthesis pathway following *Chloris virgata.* **(A)** The possible ABA signaling pathway in *C. virgata.* PYL, pyrabactin resistance like; ABI, ABA insensitive; SnRK2, sucrose non-fermenting 1 related protein kinase. **(B)** ABA biosynthesis in *C. virgata.* ABA synthesis start form carotene by oxidative cleavage reaction. BCH, beta carotenoid hydroxylase; ZEP, zeaxanthin epoxidase; NCED, 9-*cis*-epoxycratenoid dioxygenase; ABA2, ABA deficient 2; AAO, abscisic aldehyde oxidase. The arrows indicate each steps.

**FIGURE 7:**
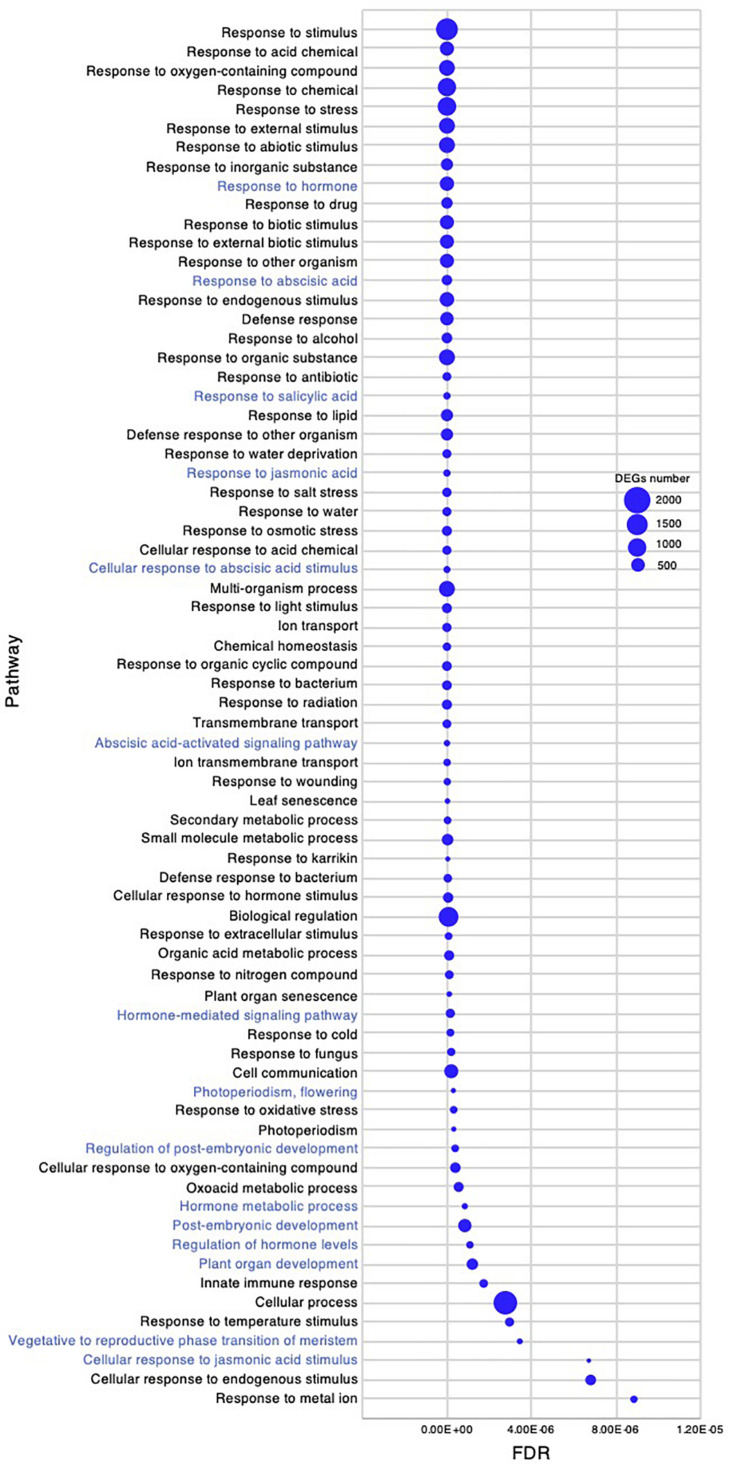
Gene ontology enrichment analysis in the early young development stage of *Chloris virgata.* Gene ontology terms of DEGs enriched in the young development stage, compared with the germination stage of *C. virgata.* False discovery rates were lower than 1.20E-05 and blue circles show gene number.

To investigate the molecular mechanism underlying the high growth performance of *C. virgata* (Figure 1), we compared the transcriptomes of samples from whole organs at the germination stage (2 DAG), early young development stage (8 DAG), young development stage (17 DAG), and adult development stage (28 DAG) (Figure 1D). Mapping the RNA-seq reads obtained by Ion Torrent sequencing to the reference transcripts, we illustrated a comprehensive gene expression map along with juvenile development in *C. virgata.* To further understand the expression changes in the four different developmental stages, we conducted GO enrichment analysis to assess overrepresented functions in the DEGs found in our pairwise comparisons of all possible sample combinations (Figure 3 and Table 2). We found that 85, 392, 77, and 31 GO terms in the biological process category were enriched (FDR < 0.03) in the germination stage, early young development stage, young development stage, and adult development stage, respectively (Figures 4–7 and Supplementary Data Sheets).

### Identification and Quantification of Phytohormone Signaling and Biosynthesis Homologous Genes of *Chloris virgata*

Phytohormones play an important role in plant development. Gibberellic acid (GA) activates and abscisic acid (ABA) suppresses plant germination. Brassinosteroids (BRs) promote shoot growth in the early young development stage and strigolactones (SLs) suppress branch formation in the adult development stage. To analyze the possible regulatory mechanisms of active germination, homologous genes of GA, ABA, BR, and SL signaling and biosynthesis were identified from the *C. virgata* unigenes database with BLASTx, using the phytohormone genes in *Arabidopsis* and rice. The expression levels of the identified GA, ABA, BR, and SL signaling and biosynthesis genes of *C. virgata* in the four independent developmental stages were analyzed using the Ion Proton Sequencer. Expression abundance was quantified based on the read count data computed using the featureCounts program and evaluated using the RPM values. The ratios of the expression levels of each gene in each development stage were calculated.

### Functional Classification of Highly Expressed Transcripts and Expressed Genes Related to GA and ABA Functioning in the Germination Stage of *Chloris virgata*

*Chloris virgata* showed faster germination performance than the other Mongolian grassland plants (Table 1), wheat, or rice (Figures 1A,B). To investigate gene expression that may be related to the germination performance of *C. virgata* (Table 1), we examined the GO terms enriched in the germination stage. We found 85 enriched categories of biological processes with FDR < 0.03 (Supplementary Data 1). As there were too many enriched GO categories to present in one figure, the 64 categories with more than 6 DEGs are presented in Figure 4, while those with fewer DEGs were removed. Several GO terms related to chloroplasts were enriched, including “chloroplast organization,” “protein targeting to chloroplast,” “establishment of protein localization to chloroplast,” “protein localization to chloroplast,” “chloroplast RNA processing,” and “chloroplast rRNA processing.” The mitochondria-related GO categories “protein import into mitochondrial intermembrane space,” “mitochondrial RNA modification,” “mitochondrial RNA metabolic process,” and “mitochondrial mRNA modification” were also enriched in this stage.

Phytohormones, GAs, and ABA antagonistically mediate several plant developmental processes, including seed maturation, seed dormancy and germination, primary root growth, and leaf development (Finkelstein et al., 2008). In particular, GA positively regulates seed germination, whereas ABA suppresses seed germination (Rajjou et al., 2012). To investigate the possible regulation of the germination stage of *C. virgata* by GA and ABA, the signaling and biosynthesis genes of GA and ABA in the *Arabidopsis* and rice genomes were used to identify homologous genes in the *C. virgata* unigenes database. Therefore, we examined the expression profiles of genes that are linked to GA and ABA signaling and biosynthesis in rice and *Arabidopsis* in the germination to early young development stage and in the young development to adult development stage of *C. virgata* (Tables 3, 4 and Supplementary Tables 1, 2). GA biosynthesis starts from the diterpenoid pathway and its formation is initiated by terpenoid cyclase (CPS) (Sun and Kamiya, 1994), and gibberellin 3-oxidase 1 (GA3-ox1). GA3-ox1 is involved in the final step of GA biosynthesis, when the active forms GA_4_ and GA_1_ are synthesized (Talon et al., 1990). Gibberrellin 2-beta-dioxygenase 1 (GA2-ox1) is metabolized from the active forms (GA_4_ and GA_1_) to inactivated GA_34_ and GA_8_ (Thomas et al., 1999; Figure 5). *CPS* (Chloris5831c000010) and *GA3-ox1* (Chloris7778c000010) homologous genes in *C. virgata* were significantly upregulated and *GA2-ox1* (Chloris20434c000010) homologs in *C. virgata* were downregulated during the germination stage (Table 3). In *Arabidopsis*, there are several protein families known as ABA receptor components, including pyrabactin resistance1 (PYR1) and pyrabactin resistance1-like proteins (PYL1) (Park et al., 2009). The plant-specific protein sucrose non-fermenting 1-related protein kinase 2 (SnRK2), mediates protein phosphorylation and the activation of the ABA signaling pathway. There are 10 members of SnRK2 in *Arabidopsis* and rice, including SnRK2B and SnRK2E (Boudsocq et al., 2004; Kobayashi et al., 2004). ABA insensitive 5 (ABI5) is a basic leucine zipper transcription factor that generally activates ABA signaling (Yu et al., 2015; Figure 6). The expression of the homologous genes of the ABA receptor component *PYL1* (Chloris6771c000010), signal transduction genes *SnRK2B* (Chloris13011c000010) and *SnRK2E* (Chloris2715c000010), and transcription factor *ABI5* (Chloris7667c000010) were downregulated in *C. virgata* during the germination stage (Table 4). In the first step in the ABA biosynthesis pathway, ABA is derived from carotene by an oxidative cleavage reaction involving 9-*cis-*epoxycarotenoid dioxygenase (NCED) in plastids (Schwartz et al., 1997; Iuchi et al., 2001; Figure 6). Expression of *NCED1* (Chloris2871c000010), *NCED2* (Chloris28063c000010), *NCED5* (Chloris11441c000010), and *NCED6* (Chloris2871c000010) was downregulated in the germination stage of *C. virgata* (Table 4).

**TABLE 3:** Gibberellin signaling and biosynthesis gene families identified from *Chloris virgata.*

Gene name	*A. thaliana*	*O. sativa*	*C. virgata*	Ratio of expression level	
				2 days/8 days	28 days/17 days
**Gibberellin signaling genes**					
GID1A GID18	At3g05120 At3g63010	Os05g0407500	Chloris4812c000010	0.569	0.910
SLY	At4g24210	Os02g0580300	Chloris1845c000010	1.110	1.295
GAi RGA RGL1	At1g14920 At2g01570 At1g66350	Os03g0707600 – Os03g0707600	Chloris3604c000010	1.501	0.678
RGL2 RGL3	At3g03450 At5g17490	Os03g0707600	Chloris25649c000010	1.046	0.990
**Gibberellin biosynthesis genes**					
CPS	At4g02780	Os02g0278700	Chloris5831c000010	5.512 ↑	0.907
KS	At1g79460	Os04g0611800	Chloris1808c000010	1.087	1.077
CYP701A3	At5g25900	Os06g0569900	Chloris20633c000010	0.852	1.091
CYP88A	At1g05160	Os06g0110000	Chloris29327c000010	0.709	1.230
			Chloris7975c000010	0.627	0.575
GA20-ox2	At5g51810	Os0190883800	Chloris27199c00001O	0.783	0.992
GA3-ox1	At1g15550	Os05g0178100	Chloris7778c000010	1.720 ↑	1.520
GA2-ox1	At1g78440	Os05g0158600	Chloris20434c000010	0.383 ↓	0.406
GA2-ox8	At4g21200	–	Chloris26078c000010	1.172	1.683

*The highest homologous gene numbers and names of Arabidopsis thaliana and Oryza sativa were aligned with C. virgata. The fold-change of gene expression in each developmental stage are shown in the right panel. Notable expression is suggested by black arrows.*

**TABLE 4:** Abscisic acid signaling and biosynthetic gene families identified from *Chloris virgata.*

Gene name	*A. thaliana*	*O. sativa*	*C. virgata*	Ratio of expression level	
				2 days/8 days	28 days/17 days
**ABA signaling genes**					
PYL1	At5g46790	Os10g0573400	Chloris6771c000010	0.861 ↓	1.173
PYL2	At2g26040	Os06g0562200	Chloris15678c000010	2.208	1.024
PYL5	At5g05440	Os03g0297600	Chloris15680c000010	1.531	0.394
SnRK28	At1g60940	Os02g0551100	Chloris13011c000010	0.533 ↓	1.043
SnRK2E	At4g33950		Chloris2715c000010	0.960 ↓	1.223
ABl1	At4g26080	Os05g0572700	Chloris27073c000010	0.715	0.986
ABl4	At2g40220	Os05g0351200	Chloris28360c000010	3.177	0.708
ABl5	At2g36270		Chloris7667c000010	0.656 ↓	1.126
**ABA biosynthesis genes**					
NCED1	At3g63520	Os12g0640600	Chloris2871c000010	0.229 ↓	1.389
NCED2	At4g18350	Os1290435200	Chloris28063c000010	0.056 ↓	1.617
NCED5	At1g30100	Os1290617400	Chloris11441c000010	0.705 ↓	0.961
NCED6	At3g24220	–	Chloris2871c000010	0.229 ↓	1.389
NCED9	At1g78390	Os03g0645900	Chloris28516c000010	1.170	0.972
ABA2	At1g52340	Os03g0810800	Chloris6223c000010	0.974	1.247
AAO2 AAO3 AAO4	At3g43600 AT2G27150 At1g04580	Os07g0164900 Os03g0790900 Os0790164900	Chloris17143c000010	1.074	0.678
CYP707A	At4g19230	Os02g0703600	Chloris18677c000010	0.741	1.160

*Possible abscisic acid homologous gene names of C. virgata are shown in the left panel. Notable expression is suggested by black arrows in the right panel.*

### Functional Classification of Highly Expressed Transcripts and Expressed Genes Related to Brassinosteroid Regulation in the Early Young Development Stage of *Chloris virgata*

*Chloris virgata* showed high growth performance with active elongation of shoots in the early young development stage, 7–8 DAG (Figures 1A,C). To investigate gene expression that may be related to the early young development stage of *C. virgata*, we examined enriched GO terms. The number of enriched GO categories in this stage was substantially higher than that in the other three development stages. A total of 392 enriched biological process categories with FDR < 0.03 were identified (Supplementary Data 2). As there were too many enriched GO categories to present in the figure, the 72 GO categories with FDR < 1.20E-05 and DEGs greater than 80 are presented in Figure 7. In the biological process category, several GO terms related to organ development were identified, including “photoperiodism, flowering,” “regulation of post-embryonic development,” “post-embryonic development,” “plant organ development,” and “vegetative to reproductive phase transition of meristem.” In the phytohormone-related GO categories, “response to hormone,” “response to ABA,” “response to ABA,” “response to salicylic acid,” “response to jasmonic acid,” “cellular response to ABA stimulus,” “ABA-activated signaling pathway,” “hormone-mediated signaling pathway,” “hormone metabolic process,” “regulation of hormone levels,” and “cellular response to jasmonic acid stimulus” were also enriched in this stage.

Brassinosteroids are phytohormones that are also categorized in a class of steroid hormones that are widely conserved from animals to plants. BRs regulate cell differentiation, cell division, and cell elongation, which together regulate the development and growth of plant leaves, stems, and roots in the young development stage (Nakano and Asami, 2014). To analyze the possible regulatory mechanisms that activate the development and growth of *C. virgata* by BR, the signaling and biosynthesis genes of BR in the *Arabidopsis* and rice genomes were used to identify homologous genes in the *C. virgata* unigenes database. Then, we examined the expression profiles of these genes in the four development stages (Table 5 and Supplementary Table 3).

**TABLE 5:** Brassinosteroid signaling and biosynthesis gene families identified from *Chloris virgata.*

Gene name	*A. thaliana*	*O. sativa*	*C. virgata*	Ratio of expression level	
				8 days/2 days	28 days/17 days
**Brassinosteroid signaling genes**					
BRl1	At4g39400	Os01g0718300	Chloris3177c000010	1.626 ↑	0.732
BRL1	At1g55610	Os08g0342300	Chloris17044c000010	0.560	1.006
BAK1	At4g33430	Os08g0174700	Chloris23503c000010	1.890 ↑	1.518
BKl1	At5g42750	Os09g0459500	Chloris17074c000010	1.161	0.964
BSK1	At4g35230	Os03g0132800	Chloris3139c000010	1.541 ↑	1.008
BSU1	At1g03445	Os05g0144400	Chloris13780c000010	1.720 ↑	1.157
BIN2	At4g18710	Os05g0207500	Chloris6135c000010	0.876	1.099
BSS1	At3g57130	Os01g0948900	Chloris13348c000010	0.704 ↓	1.060
BIL1/BZR1 BES1	At1g75080 At1g19350	Os07g0580500	Chloris30620c000010	0.731	1.312
**Brassinosteroid biosynthesis genes**					
DET2	At2g38050	Os01g0851600	Chloris12539c000010	1.563 ↑	1.197
DWF4	At3g50660	Os03g0227700	Chloris405c000010	1.174 ↑	0.369
CPD	At5g05690	Os11g0143200	Chloris6902c000010	1.231 ↑	0.999
BR6ox	At5g38970	Os03g0602300	Chloris14424c000010	1.060 ↑	1.509
BAS1	At2g26710	Os02g0537700	Chloris30576c000010	0.310 ↓	1.542

*Possible positive signaling genes and biosynthesis genes of brassinosteroid were more highly expressed in the early young development stage (8 days) than in the germination stage (2 days). Notable expression is suggested by black arrows in the right panel.*

**FIGURE 8:**
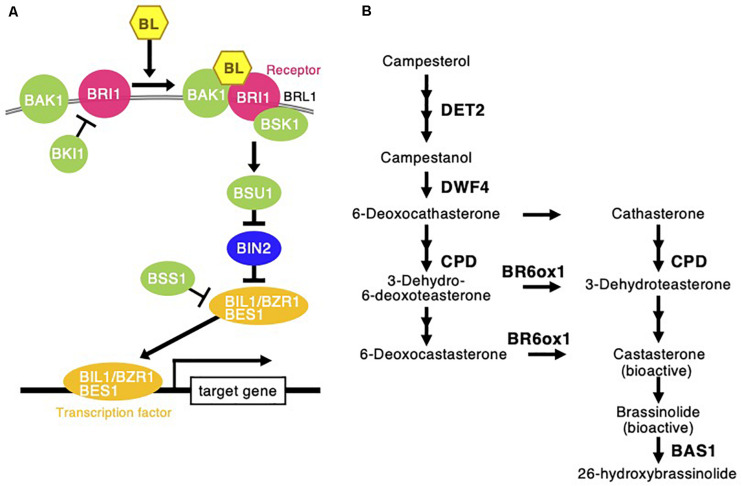
Brassinosteroid signaling and biosynthesis pathway following *Chloris virgata*. **(A)** The possible BR signaling transduction in *C. virgata.* BAK1, BRI1 associated receptor kinase; BRI1, BR insensitive 1; BRL1, BRI1 like; BSK1, BR signaling kinase 1; BKI1, BRI1 kinase inhibitor 1; BSU1, BRI1 suppressor 1; BIN2, BR insensitive 2; BSS1, blade on petiole 1; BES1, brassinazole resistant 2; BZR1, brassinazole resistant 1. **(B)** BR biosynthesis early C-22 oxidation and late C-6 oxidation pathway in *C. virgata.* DET2, de-etiolated 2; DWF4, dwarf 4; CPD, constitutive photomorphogenic dwarf; BR6ox, BR 6-oxidase; BL, brassinolide; BAS1, phyb activation tagged suppressor 1. The arrows indicate each steps.

Brassinosteroids are accepted by plasma membrane protein complexes formed by the BR-type transmembrane receptor kinase BR insensitive 1 (BRI1) (Li and Chory, 1997), BRI1-associated receptor kinase (BAK1) (Li et al., 2002), and membrane-associated BR-signaling kinase 1 (BSK1) (Tang et al., 2008) as positive factors for BR signaling (Figure 8). The expression of these three positive BR receptor complex protein homologs in *C. virgata* (*BRI*: Chloris3177c000010, *BAK1*: Chloris23503c000010, and *BSK1*: Chloris3139c000010) was upregulated in the early young development stage of *C. virgata* (Table 5). In the middle stream of BR signaling, BRI1 suppressor 1 (BSU1) is a positive phosphatase (Mora-García et al., 2004) and brassinozole-sensitive-short hupocotyl 1 (BSS1) is a negative regulator of the BR master transcription factor brassinozole-insensitive-long hupocotyl 1 (BIL1) (Jun et al., 2010; Figure 8). Expression of the *C. virgata BSU1* homolog (Chloris13780c000010) was upregulated and that of the *BSS1* homolog (Chloris13348c000010) was downregulated at this stage. BR biosynthesis plays an important role in the activation of BR signaling (Table 5).

De-etiolated 2 (DET2) is a homolog of steroid 5 α-reductase in humans. It works upstream of BR biosynthesis (Li et al., 1996). Dwarf 4 (DWF4) (Choe et al., 1998) and constitutive photomorphogenesis and dwarfism (CPD) (Szekeres et al., 1996) are major P450 oxidase in the intermediate step of BR biosynthesis. BR C-6 oxidase (CYP85A1) is also P450 oxidase in the last step of biosynthesis (Shimada et al., 2001). PhyB activation-tagged suppressor 1 (BAS1) metabolizes active brassinolide to non-active 26-hydroxybrassinolide (Neff et al., 1999; Figure 8). Expression of the *C. virgata* homologous genes of *DET2* (Chloris12539c000010), *DWF4* (Chloris405c000010), *CPD* (Chloris6902c000010), and *BR6OX* (Chloris14424c000010) were upregulated and that of *BAS1* (Chloris30576c000010) was downregulated in the early young development stage of *C. virgata* (Table 5).

### Functional Classification of Highly Expressed Transcripts and Expressed Genes Related to Strigolactone Functioning in the Adult Development Stage of *Chloris virgata*

**FIGURE 9:**
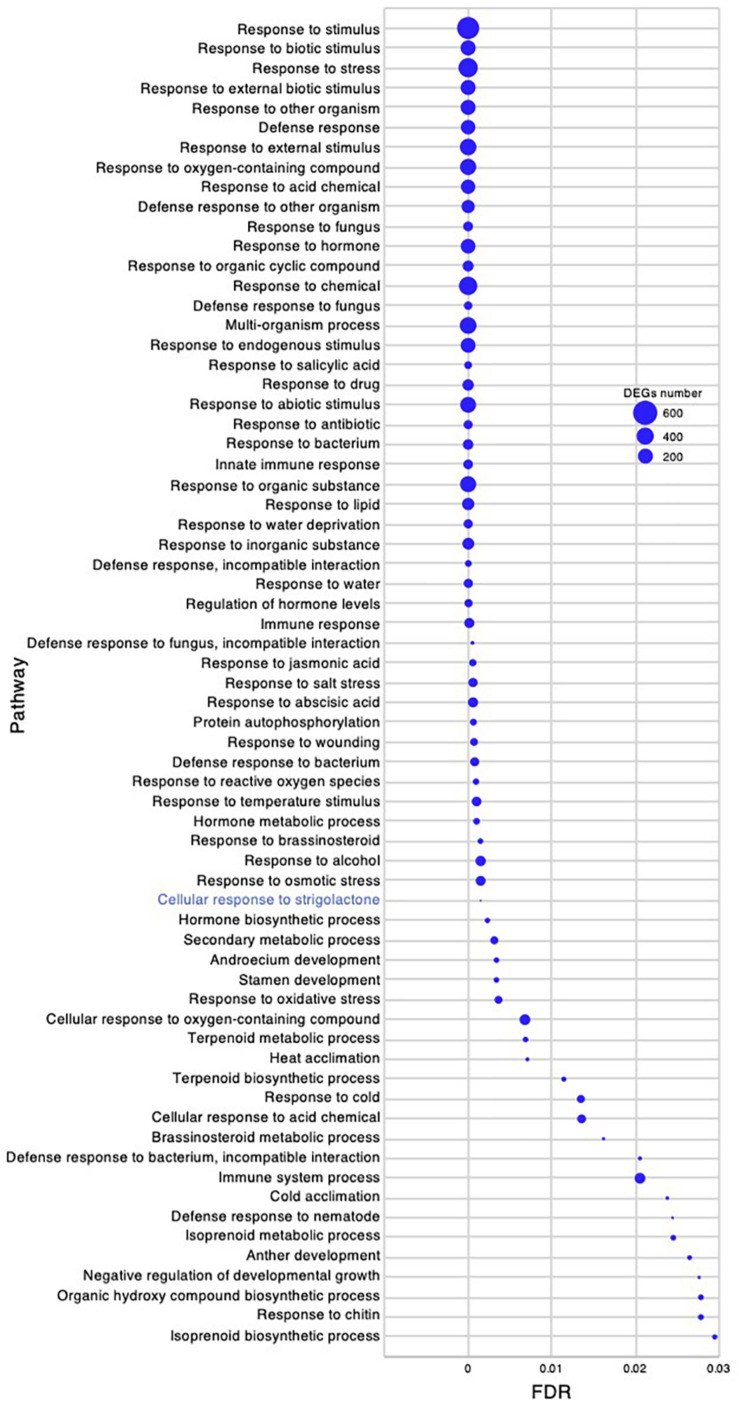
Gene ontology enrichment analysis in the young development stage of *Chloris virgata* grown on soil. Gene ontology terms of DEGs enriched in the young development stage (17 days after germination), compared with the adult development stage (28 days after germination) of *C. virgata*. False discovery rates were lower than 0.03 and blue circles show gene number.

The active branch formation of *C. virgata* started from the young development stage and continued throughout the adult development stage to the flowering stage. Increased branch formation during animal bite-mimicking cutting experiment was also observed (Figures 1D, 2). These observations suggest that *C. virgata* possesses a high branch formation potential. To investigate gene expression that may be related to high branch formation in *C. virgata*, we examined the GO terms in the young and adult developmental stages. A total of 77 biological process GO categories were enriched in the young development stage (FDR < 0.03) (Supplementary Data 3). As there were too many enriched GO categories in the young development stage to present in the figure, the 67 categories with FDR < 0.03 and DEGs > 10 are presented in Figure 9, including “cellular response to SL.”

**FIGURE 10:**
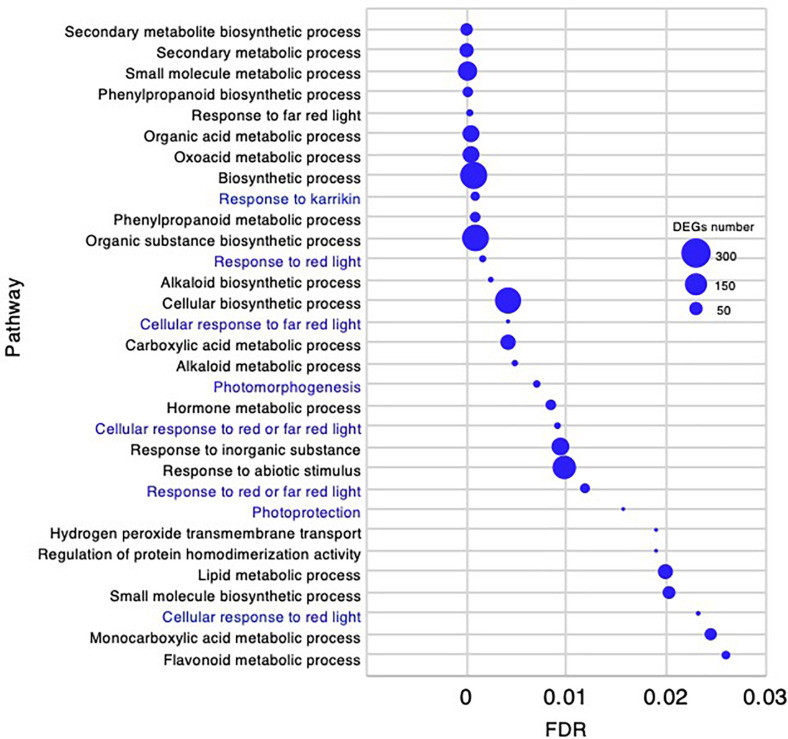
Gene ontology enrichment analysis in the adult development stage of *Chloris virgata* grown on soil. Gene ontology terms of DEGs enriched in the adult development stage compared with the young development stage of *C. virgata*. False discovery rates were lower than 0.03 and blue circles show gene number.

In the adult development stage, 31 biological processes GO categories were enriched and had FDR < 0.03 (Supplementary Data 4). As there were fewer enriched GO categories than at the other stages, all 31 are presented in Figure 10. Of the enriched categories, the response to abiotic stimulus-related GO terms could be characteristically identified as “response to karrikin,” “response to red light,” “cellular response to far red light,” “cellular response to red light,” and “photomorphogenesis.”

**FIGURE 11:**
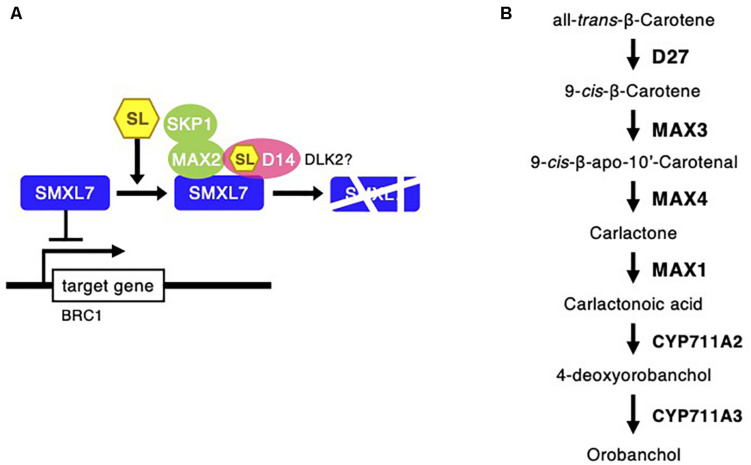
Strigolactone signaling and biosynthesis pathway following *Chloris virgata*. **(A)** The possible SLs signaling pathway in *C. virgata*. D14, dwarf 14; DLK2, D14 like 2; MAX2, more axillary branches 2; SKP1, S phase kinase-associated protein 1; SMXL7, suppressor of MAX2 1 like 7; BRC1, branched 1. **(B)** SLs biosynthesis pathway in *C. virgata.* D27, dwarf 27. The arrows indicate each steps.

Strigolactones are phytohormones known to suppress plant branch formation (Umehara et al., 2008), and biosynthesis-deficient mutants have shown an increased number of tillers in rice (Ishikawa et al., 2005). To analyze the possible SL-related regulatory mechanism for the high branch formation of *C. virgata*, the signaling and biosynthesis genes of SLs in *Arabidopsis* and rice were used to identify homologous genes in the *C. virgata* unigenes database and the expression level of each gene in each development stages were calculated (Supplementary Table 4). DWARF14 (D14) has been identified as a receptor of SLs in *Arabidopsis* and rice (Arite et al., 2009), and DWARF14-LIKE2 (DLK2) also acts as an SL receptor. MORE AXILLARY GROWTH 2 (MAX2) forms a protein complex with D14 as a core signaling factor in the SL signaling pathway (Stirnberg et al., 2002; Végh et al., 2017). BRANCHED1 (BRC1) is a transcription factor that acts as a suppressor of branching (Aguilar-Martínez et al., 2007; Finlayson, 2007). Knock out of the SL receptors, MAX2 and BRC1, causes drastic promotion of branch formation in plants (Stirnberg et al., 2002; Aguilar-Martínez et al., 2007; Figure 11). The expression of the *C. virgata* homologous genes of *D14* (Chloris9047c000010), *DLK2* (Chloris3102c000010; Chloris16862c000010; Chloris16863c00010), *MAX2* (Chloris21103c000010), and *BRC1* (Chloris9240c000010) were downregulated in the adult developmental stage of *C. virgata* (Table 6).

**TABLE 6:** Strigolactones signaling and biosynthesis gene families identified from *Chloris virgata*.

Gene name	*A. thaliana*	*O. sativa*	*C. virgata*	Ratio of expression level	
				8 days/2 days	28 days/17 days
**Strigolactones signaling genes**					
D14	AT3G03990	Os03g0203200	Chloris9047c000010	5.382	0.614 ↓
DLK2	At3g24420	Os05t0590399	Chloris3102c000010	1.207	0.793 ↓
		Os01t0595600	Chloris16862c000010	0.667	0.359 ↓
			Chloris16863c000010	1.384	0.395 ↓
MAX2	AT2G42620	Os06g0154200	Chloris21103c000010	0.768	1.015 ↓
SKP1	At1g75950	Os11g0456300	Chloris20528c000010	1.341	1.211
BRC1	AT3G18550	Os09g0410500	Chloris9240c000010	2.542	1.050 ↓
SMXL7	AT2G29970	–	Chloris14785c000010	2.662	1.608
**Strigolactones biosynthesis genes**					
D27	AT1G03055	–	Chloris8648c000010	1.047	1.254
MAX3	At2g44990	Os04g0550600	Chloris8839c000010	0.773	1.307
MAX4	At4g32810	Os01g0746400	Chloris11425c000010	0.970	1.975
			Chloris20143c000010	1.839	2.299
MAX1	At2g26170	Os01g0700900	Chloris23859c000010	1.079	0.797
CYP711A2 CYP711A3	-	Os01g0700900 Os0190701400	Chloris16015c000010	0.949	1.492

*Possible strigolactone homologous genes of C. virgata are shown in the left panel. Notable expression is suggested by black arrows in the right panel.*

## Discussion

In this study, we identified the *C. virgata* DG accession as the fastest germination plant by seed screening 40 major Mongolian grassland plants. *C. virgata* also showed faster germination than the common agricultural crops, rice and wheat (Table 1). *C. virgata* is one of the major plants in the Mongolian grassland. There are approximately 50 species of *Chloris* that grow in most tropical areas worldwide, but only one species, *C. virgata*, has been found in Mongolia (Jigjidsuren and Johnson, 2003). *Chloris gayana* is a popular grass in pasture land, and its salt tolerance mechanism is to excrete salt via salt glands (Liphschitz et al., 1974; Kobayashi et al., 2007). *C. virgata* Sw., known as feather finger grass, is an annual grass of the Gramineae family, which is widespread across many habitats. The general phenotype is 5–50 cm tall, with semi-prostrate stems with 3–4 nodes; linear lanceolate leaflets, 5–15 cm long and approximately 2 mm wide; and arrowhead-type seeds, 2–3 mm long, with seed covers with long white hairs that can be carried by wind or water (Jigjidsuren and Johnson, 2003).

Previous research on *C. virgata* has been limited to a few fields based on specific ecological or physiological aspects. *C. virgata* is thought to be one of the major C_4_ summer grass weeds in Australia. It was found in 118,000 hectares of Australian crop farms, and a trial to decrease *C. virgata* growth using herbicide has been reported (Davidson et al., 2019; Mobli et al., 2020). *C. virgata* also tends to be resistant to alkaline soil conditions (Nishiuchi et al., 2010). Characteristics of *C. virgata* might suggest that it possesses tough viability, but direct research on its growth characteristics has been limited (Yang et al., 2008; Lin et al., 2016; Zhong et al., 2017).

Nevertheless, a previous report analyzed the phenotype and composition of fundamental components in the germination stage of six grassland species in Northeast China: *C. virgata, Kochia scoparia, Lespedeza hedysaroides, Astragalus adsurgens, Leonurus artemisia*, and *Dracocephalum moldavica* (Zhao et al., 2018). The report suggests that *C. virgata* started germination 10 h after imbibition, which was faster than the other four grassland plants. As we found that *C. virgata* germination started 5 h after germination treatment, these results were similar in both Mongolia and Northeast China. Furthermore, *C. virgata* quickly absorbed water during the first 2 h of imbibition, reached 73% water absorption. Dry *C. virgata* seeds contained approximately 62.2% starch, which was the highest ratio among the six grassland plants. Soluble sugars quickly increased after *C. virgata* germination. This starch composition might be one of the adaptations supporting the fast germination potential of *C. virgata* (Zhao et al., 2018).

When the apical meristem of the main stem in dicots and monocots is cut off, elongation of the lateral bud is generally observed, and these mechanisms are based on the phytohormones auxin and cytokinin as an apical dominance system (Domagalska and Leyser, 2011). In nomadic areas, all plants are exposed to the risk of animal bite. Several plants might die after a single animal bite, but other plants might be able to survive in this risky environment. In our survey of nomadic culture and previous research, *C. virgata* was shown to possess high regrowth ability after animal bite (Jigjidsuren and Johnson, 2003). We attempted to confirm the possible restoration power of *C. virgata.* As shown in Figure 2, *C. virgata* showed high regrowth potential after scissor cutting that mimicked animal bite, when compared with wheat and rice. The number of regrowth shoots of wheat and rice decreased with increased cutting, but *C. virgata* still maintained the ability to regrow after six cutting treatments. In a previous study, *Medicago truncatula* were cut off at 25 and 75% of the main and lateral shoots at the early (80 days old) and late (140 days old) development stages, and growth parameters such as shoot biomass and branch number were measured. The shoot biomass and branching number of *M. truncatula* showed recovery after 25% cutting during the early stage, but plants subjected to 75% cutting could not recover to the height they were before the treatment. Furthermore, the *M. truncatula* analysis was performed only once, and the phenotype after a second cutting has not been reported (Gruntman and Novoplansky, 2011). The analysis of plant growth after animal bite in grassland ecosystems has been performed in the field. Regrowth potential analysis of a *Hordeum brevisubulatum* population was conducted using three different cutting treatments (light cutting with a stubble height of 15 cm, medium cutting with a stubble height of 10 cm, and heavy cutting with a stubble height of 5 cm) and regrowth potential was calculated 1, 3, and 7 weeks after cutting. Heavily cut *H. brevisubulatum* showed regrowth to 25% of the height of an uncut plant 3 weeks after cutting and to 80% of that height 7 weeks after cutting (Yuan et al., 2020). In our study, *C. virgata* still showed regrowth to almost the same plant height after the sixth cutting. Our results indicating the regrowth advantages of *C. virgata* might contribute to the recovery of grasslands under severe stress from livestock husbandry in Mongolia.

In previous research, a few molecular biology studies have been performed and reported. Glyphosate is one of the strongest herbicides that binds to the enzyme 5-enolpyruvylshikimate-3-phosphate synthase (EPSPS) and inhibits amino acid metabolism in the shikimate pathway (Amrhein et al., 1980). In Australian grasslands, *C. virgata* showed resistance against glyphosate, which evolved and increased. Sequencing analysis of the EPSPS gene of the glyphosate-resistant *C. virgata* suggested several amino acid mutations at the herbicide target site (Ngo et al., 2018). In a previous study, 3168 expressed sequence tags (ESTs) were selected and sequenced from a cDNA library of NaHCO_3_-treated *C. virgata*, based on seeds originally collected in North China (Nishiuchi et al., 2010). Of these, 2590 ESTs indicated similarity to sequences in the NCBI database, and about 67% of the unigenes were annotated to genes in the rice genome. GO analysis revealed that 1,081 genes were annotated and distributed among 1245 terms in biological process, 1126 terms in molecular function, and 1415 terms in cellular component. In the GO functional categories for EST, 75 genes were annotated with the GO term “response to stress,” which might be owing to the experimental conditions, as these ESTs were identified from NaHCO_3_-treated *C. virgata.* A previous study also showed that the *MT1* gene was upregulated in *C. virgata* under saline–alkaline conditions and heavy metal stress. Yeasts transformed with the *MT1a* gene of *C. virgata* showed tolerance to alkali stress. The *ChvACT2* in *C. virgata* mRNA was upregulated more than twofold under alkali stress, such as that caused by NaCl, NaHCO_3_, CuSO_4_, ZnSO_4_, and CoCl_2_ (Nishiuchi et al., 2010). These reports are pioneering studies on the molecular biology of *C. virgata.* Based on the interesting phenotype of *C. virgata* as the fastest-growing plant, we began collecting more detailed genomic information.

In this study, transcriptome analyses of *C. virgata* at the germination (2 DAG), early development (8 DAG), young development (17 DAG), and adult development (28 DAG) stages were revealed. The cDNA library of these four groups of *C. virgata* yielded 21,589 contigs that were predicted to ORFs. Of these, 19,346 and 18,156 ORFs were matched to homologous genes in *O. sativa* and *A. thaliana*, respectively (Table 2).

In the germination stage of *C. virgata*, of the 85 enriched GO terms, six chloroplast- and four mitochondria-related GO terms were enriched (Figure 4 and Supplementary Data 1). A previous transcriptome analysis of the germination process of rice revealed that the GO terms involving chloroplasts and mitochondria were enriched 24 h after germination (Howell et al., 2009). Photosynthesis- and mitochondria-related proteins are also abundant in wheat and barley (Sreenivasulu et al., 2008; Yu et al., 2014). These results are similar to those of *C. virgata*. Furthermore, lipid metabolism-related GO terms were also enriched in the germination stages of *C. virgata* and rice. These systems are quickly induced and prepared to perform seed germination.

Plant germination is tightly regulated by phytohormone associations, GA, and ABA. In particular, GA and ABA play key roles in the germination stage and in the control of seed dormancy respectively. In this study, the ABA biosynthesis enzyme (NCED) family homologous genes (*NCED1*: Chloris2871c000010, *NCED2*: Chloris28063c000010, *NCED5*: Chloris11441c000010, and *NCED6*: Chloris2871c000010), regulatory component of the ABA receptor (RCAR) family homologous gene (*PYL1*: Chloris6771c000010), ABA signal transduction-related kinase SnRK2 homologous genes (*SnRK2B*: Chloris13011c000010 and *SnRK2E*: Chloris2715c000010), and basic leucine zipper (bZIP) transcription factor homologous gene (*ABI5*: Chloris7667c000010) were downregulated during the germination stage of *C. virgata* (Table 4). In previous studies, NCED5- and NCED6-deficient mutants showed higher germination efficiency than wild-type *Arabidopsis* (Frey et al., 2012). Our results show that the GA biosynthesis genes (*CPS*: Chloris5831c000010 and *GA3-ox1*: Chloris7778c000010) were significantly upregulated during the germination stage of *C. virgata* (Table 3). In *Arabidopsis*, GA3-ox1-deficient mutants were non-germinating until exogenous GA supplementation (Ogawa et al., 2003). Additionally, the mRNA expression of the GA deactivation enzyme *AtGA2ox* was extremely low during the early germination stage of *Arabidopsis* (Ogawa et al., 2003). Our results show that one of the GA deactivation enzymes, *GA2-ox1* homologous gene (Chloris20434c000010), was expressed at extremely low levels during the germination stage of *C. virgata* (Table 3). The changes in the expression of ABA- and GA-related genes in the germination stage of *C. virgata* might be involved in the fast germination rate of *C. virgata.*

As shown in Figures 1A,C, *C. virgata* showed an active leaf elongation phenotype in the early developmental stage. At this stage, 392 biological process GO categories, including many organ development promotion-related categories, were overrepresented and enriched (Figure 7 and Supplementary Data 2). Based on these plant growth categories, the homologs of the biosynthesis enzyme of the plant growth and development promoting hormone BR (*DET2*: Chloris12539c000010, *DWF4*: Chloris405c000010, *CPD*: Chloris6902c000010, and *BR6OX*: Chloris144424c000010) were upregulated in the early young development stage of *C. virgata* (Table 5). In the early stages of BR research, the first BR biosynthesis gene (*det2*) mutants were isolated as dwarf phenotypes in *Arabidopsis* (Li et al., 1996; Szekeres et al., 1996). Furthermore, mutants deficient in the biosynthesis genes DWF4 and CPD showed dwarfism and were rescued by exogenously supplied BR (Szekeres et al., 1996; Fujiyama et al., 2019). Conversely, the BR catabolic enzyme *BAS1* homologous gene (Chloris30576c000010) was downregulated in the early young development stage of *C. virgata* (Table 5). Overexpression of BAS1 caused dwarfism and decreased the interval content of brassinolide in *Arabidopsis* (Neff et al., 1999). Furthermore, the homologous genes of the transmembrane receptors of BR on the plasma membrane, *BRI1* (Chloris3177c000010), *BAK1* (Chloris23503c000010), and BR-signaling kinase, *BSK1* (Chloris3139c000010), were upregulated in the early young development stage of *C. virgata* (Table 5). BRI1 is the major receptor of BR signaling, and mutations in BRI1 cause extreme dwarfism and cannot be rescued by exogenous BL (Wang et al., 2001; Caño-Delgado et al., 2004). BRI1 overexpression promotes the organ growth phenotype as an exogenous BR treatment (Wang et al., 2001). Overexpression of BAK1 promoted leaf growth, and BAK1-deficient mutants showed a semi-dwarfed phenotype that also exhibited reduced sensitivity to BR treatment (Li et al., 2002). BSK1 is a membrane-associated kinase, and overexpression of BSK1 rescued the dwarf phenotype of the BR-insensitive *bri1* mutant (Tang et al., 2008). The BR-signaling positive phosphatase, *BSU1*, (Chloris13780c000010) homolog was upregulated in the early young development stage of *C. virgata* (Table 5). Overexpression of *BSU1* rescued the *bri1* dwarf phenotype (Mora-García et al., 2004). A negative regulator of the BR-signaling factor, *BSS1*, homologous gene (Chloris13348c000010) was downregulated in the early young development stage of *C. virgata* (Table 5). The *bss1-1D* mutant, which overexpressed *BSS1*, exhibited a BR biosynthesis inhibitor (Brz)-hypersensitive phenotype in hypocotyl elongation and exhibited no nuclear transfer of the BR master transcription factors, BIL1/BZR1 (Shimada et al., 2015). These results indicate that BR biosynthesis and signaling could promote the fast growth of *C. virgata* in the early young development stage.

In the adult development stage, *C. virgata* promoted branch formation (Figure 1D). Plant branch formation is negatively regulated by the phytohormone SL, which suppresses branch formation (Umehara et al., 2008). In the present study, the SL signaling homologous genes (*D14*: Chloris9047c000010; *DLK2*: Chloris3102c000010, Chloris16862c000010, and Chloris16863c000010; *MAX2*: Chloris21103c000010; and *BRC1*: Chloris9240c000010) were downregulated in the adult development stage (Table 6). DWARF14 and D14 have been identified as SL receptors, and *d14-*deficient mutants exhibit increased branching in rice (Arite et al., 2009). DLK2 is a divergent member of the D14 family that weakly binds or hydrolyzes SL ligands, and detailed regulations are still unclear in adult plants (Végh et al., 2017). The F-box leucine-rich repeat family protein MAX2 is a positive regulator of SL signaling and binds to the SL receptor D14. MAX2 is required to repress bud outgrowth at each node (Stirnberg et al., 2007). BRC1 is a positive transcription factor in SL signaling that is expressed in developing buds and leads to the suppression of bud development. The downregulation of BCR1 results in a high branching phenotype in *Arabidopsis* (Aguilar-Martínez et al., 2007). These results suggest that the downregulation of SL signaling gene expression promotes branch formation in the adult development stage of *C. virgata*.

Our report suggests that *C. virgata* possesses fast growth and high regrowth potential. Recently, Mongolian grasslands have been exposed to grazing pressure by the increasing number of livestock, such as sheep, goats, horses, cattle, camels, and other domestic animals. Climate change owing to global warming has also impacted and decreased the area of Mongolian grassland. We hope that the possible growth activity of *C. virgata* illustrated in this study will contribute to improving the robustness of the Mongolian grassland. Furthermore, we found that novel functional genes, including phytohormone signaling and biosynthesis genes, contribute to the rapid growth of *C. virgata.* A more detailed analysis of the novel genes of *C. virgata* could suggest possible functions in the improvement of growth and rapid germination in commercial crops, such as rice and wheat, and in Mongolian grassland plants.

## Data Availability Statement

The datasets presented in this study can be found in online repositories. The names of the repository/repositories and accession number(s) can be found below: https://www.ddbj.nig.ac.jp/, DRA011714.

## Author Contributions

TN, AY, JB, B-OD, TA, and KS conceived and designed the experiments. BB, AY, and SK performed the experiments, phytohormone analog search, and phylogenetic analysis. GU, SJ, and TB performed the seed germination and seed collection. KM, FT, KoI, AK, MK, and KeI performed the sequencing and *de novo* transcriptome analyses. BB, AY, FT, KM, and TN contributed to the manuscript preparation. All the authors contributed to the article and approved the submitted version.

## Conflict of Interest

The authors declare that the research was conducted in the absence of any commercial or financial relationships that could be construed as a potential conflict of interest.

## Footnotes

1 transdecoder.github.io/

2 http://github.com/iontorrent/tmap

## References

[B1] Aguilar-Martínez J. A. Poza-Carrión C. Cubas P. (2007). *Arabidopsis* Branched1 acts as an integrator of branching signals within axillary buds. *Plant Cell* 19 458–472. 10.1105/tpc.106.048934 17307924 PMC1867329

[B2] Amrhein N. Deus B. Gehrke P. Steinrücken H. C. (1980). The site of the inhibition of the shikimate pathway by glyphosate. *Plant Physiol.* 66 830–834. 10.1104/pp.66.5.830 16661535 PMC440735

[B3] Anders S. Pyl P. T. Huber W. (2015). HTSeq-A Python framework to work with high-throughput sequencing data. *Bioinformatics* 31 166–169. 10.1093/bioinformatics/btu638 25260700 PMC4287950

[B4] Arite T. Umehara M. Ishikawa S. Hanada A. Maekawa M. Yamaguchi S. (2009). D14, a strigolactone-Insensitive mutant of rice, shows an accelerated outgrowth of tillers. *Plant Cell Physiol.* 50 1416–1424. 10.1093/pcp/pcp091 19542179

[B5] Bolger A. M. Lohse M. Usadel B. (2014). Trimmomatic: a flexible trimmer for Illumina sequence data. *Bioinformatics* 30 2114–2120. 10.1093/bioinformatics/btu170 24695404 PMC4103590

[B6] Boudsocq M. Barbier-Brygoo H. Laurière C. (2004). Identification of nine sucrose nonfermenting 1-related protein kinases 2 activated by hyperosmotic and saline stresses in *Arabidopsis thaliana*. *J. Biol. Chem.* 279 41758–41766. 10.1074/jbc.M405259200 15292193

[B7] Caño-Delgado A. Yin Y. Yu C. Vefeados D. Mora-García S. Cheng J. C. (2004). BRL1 and BRL3 are novel brassinosteroid receptors that function in vascular defferentiation in *Arabidopsis*. *Development* 131 5341–5354. 10.1242/dev.01403 15486337

[B8] Choe S. Dilkes B. P. Fujioka S. Takatsuto S. Sakurai A. Feldmann K. A. (1998). The DWF4 gene of *Arabidopsis* encodes a cytochrome P450 that mediates multiple 22α-hydroxylation steps in brassinosteroid biosynthesis. *Plant Cell* 10 231–243. 10.1105/tpc.10.2.231 9490746 PMC143988

[B9] Davidson B. Cook T. Chauhan B. S. (2019). Alternative options to glyphosate for control of large echinochloa colona and *Chloris virgata* plants in cropping fallows. *Plants* 8 245–256. 10.3390/plants8080245 31344913 PMC6724109

[B10] Domagalska M. A. Leyser O. (2011). Signal integration in the control of shoot branching. *Nat. Rev. Mol. Cell Biol.* 12 211–221. 10.1038/nrm3088 21427763

[B11] Emms D. M. Kelly S. (2019). OrthoFinder: phylogenetic orthology inference for comparative genomics. *Genome Biol.* 20 238–252. 10.1186/s13059-019-1832-y 31727128 PMC6857279

[B12] Finkelstein R. Reeves W. Ariizumi T. Steber C. (2008). Molecular aspects of seed dormancy. *Annu. Rev. Plant Biol.* 59 387–415. 10.1146/annurev.arplant.59.032607.092740 18257711

[B13] Finlayson S. A. (2007). *Arabidopsis* TEOSINTE BRANCHED1-LIKE 1 regulates axillary bud outgrowth and is homologous to monocot TEOSINTE BRANCHED1. *Plant Cell Physiol.* 48 667–677. 10.1093/pcp/pcm044 17452340

[B14] Frey A. Effroy D. Lefebvre V. Seo M. Perreau F. Berger A. (2012). Epoxycarotenoid cleavage by NCED5 fine-tunes ABA accumulation and affects seed dormancy and drought tolerance with other NCED family members. *Plant J.* 70 501–512. 10.1111/j.1365-313X.2011.04887.x 22171989

[B15] Fu L. Niu B. Zhu Z. Wu S. Li W. (2012). CD-HIT: accelerated for clustering the next-generation sequencing data. *Bioinformatics* 28 3150–3152. 10.1093/bioinformatics/bts565 23060610 PMC3516142

[B16] Fujiyama K. Hino T. Kanadani M. Watanabe B. Jae Lee H. Mizutani M. (2019). Structural insights into a key step of brassinosteroid biosynthesis and its inhibition. *Nat. Plants* 5 589–594. 10.1038/s41477-019-0436-6 31182839

[B17] Gordo S. M. C. Pinheiro D. G. Moreira E. C. O. Rodrigues S. M. Poltronieri M. C. de Lemos O. F. (2012). High-throughput sequencing of black pepper root transcriptome. *BMC Plant Biol.* 12:168. 10.1186/1471-2229-12-168 22984782 PMC3487918

[B18] Grabherr M. Haas B. Yassour M. Levin J. Thompson D. Amit I. (2011). Trinity: reconstructing a full-length transcriptome without a genome from RNA-Seq data. *Nat. Biotechnol.* 29 644–652. 10.1038/nbt.1883.Trinity 21572440 PMC3571712

[B19] Grubov V. I. (2008). *Key to the vascular plants of Mongolia.* Mongolia: Gan print.

[B20] Gruntman M. Novoplansky A. (2011). Ontogenetic contingency of tolerance mechanisms in response to apical damage. *Ann. Bot.* 108 965–973. 10.1093/aob/mcr204 21873259 PMC3177681

[B21] Hereward J. P. Werth J. A. Thornby D. F. Keenan M. Chauhan B. S. Walter G. H. (2016). Complete chloroplast genome sequences of two species of *Chloris grass*, *Chloris truncata* Sw. and *Chloris virgata* R. *Br. Mitochondr. DNA Part B Resour.* 1 960–961. 10.1080/23802359.2016.1266705 33473692 PMC7799513

[B22] Hoang D. T. Chernomor O. Von Haeseler A. Minh B. Q. Vinh L. S. (2018). UFBoot2: improving the ultrafast bootstrap approximation. *Mol. Biol. Evol.* 35 518–522. 10.1093/molbev/msx281 29077904 PMC5850222

[B23] Howell K. A. Narsai R. Carroll A. Ivanova A. Lohse M. Usadel B. (2009). Mapping metabolic and transcript temporal switches during germination in rice highlights specific transcription factors and the role of RNA instability in the germination process. *Plant Physiol.* 149 961–980. 10.1104/pp.108.129874 19074628 PMC2633829

[B24] Huang X. Wang J. Aluru S. Yang S. Hillier L. (2003). PCAP: a whole-genome assembly program. *Genome Res.* 13 2164–2170. 10.1101/gr.1390403.1 12952883 PMC403719

[B25] Ishikawa S. Maekawa M. Arite T. Onishi K. Takamure I. Kyozuka J. (2005). Suppression of tiller bud activity in tillering dwarf mutants of rice. *Plant Cell Physiol.* 46 79–86. 10.1093/pcp/pci022 15659436

[B26] Iuchi S. Kobayashi M. Taji T. Naramoto M. Seki M. Kato T. (2001). Regulation of drought tolerance by gene manipulation of 9-cis-epoxycarotenoid dioxygenase, a key enzyme in abscisic acid biosynthesis in *Arabidopsis*. *Plant J.* 27 325–333. 10.1046/j.1365-313X.2001.01096.x 11532178

[B27] Jamyandorj J. Ligaa U. Otgonbileg K. Saaral N. (2011). *Very Rare, Rare and Important Useful Plants, Cultivating in Khuduu-Aral of Kherlen.* Ulaanbaatar: Ulaanbaatar Print.

[B28] Jigjidsuren S. Johnson D. A. (2003). *Forage Plants in Mongolia.* Ulaanbaatar: Admon printing.

[B29] Johnson L. S. Eddy S. R. Portugaly E. (2010). Hidden Markov model speed heuristic and iterative HMM search procedure. *BMC Bioinformatics* 11:431. 10.1186/1471-2105-11-431 20718988 PMC2931519

[B30] Jun J. H. Ha C. M. Fletchera J. C. (2010). BLADE-ON-PETIOLE1 coordinates organ determinacy and axial polarity in *Arabidopsis* by directly activating ASYMMETRIC LEAVES2. *Plant Cell* 22 62–76. 10.1105/tpc.109.070763 20118228 PMC2828709

[B31] Kalyaanamoorthy S. Minh B. Q. Wong T. K. F. Von Haeseler A. Jermiin L. S. (2017). ModelFinder: fast model selection for accurate phylogenetic estimates. *Nat. Methods* 14 587–589. 10.1038/nmeth.4285 28481363 PMC5453245

[B32] Karban R. Baldwin I. T. (1997). *Induced Responses to Herbivory.* Chicago: University of Chicago Press.

[B33] Katoh K. Standley D. M. (2013). MAFFT multiple sequence alignment software version 7: improvements in performance and usability. *Mol. Biol. Evol.* 30 772–780. 10.1093/molbev/mst010 23329690 PMC3603318

[B34] Kobayashi H. Masaoka Y. Takahashi Y. Ide Y. Sato S. (2007). Ability of salt glands in Rhodes grass (*Chloris gayana* Kunth) to secrete Na+ and K+. *Soil Sci. Plant Nutr.* 53 764–771. 10.1111/j.1747-0765.2007.00192.x

[B35] Kobayashi Y. Yamamoto S. Minami H. Kagaya Y. Hattori T. (2004). Differential activation of the rice sucrose nonfermenting1-related protein kinase2 family by hyperosmotic stress and abscisic acid. *Plant Cell* 16 1163–1177. 10.1105/tpc.019943 15084714 PMC423207

[B36] Lapin K. Eipeldauer A. Folly G. Mank D. Bernhardt K. (2017). The vegetation of North-Western mongolia: floristic checklist and conservation status of mongolian grassland flora. *Mongolian J. Biol. Sci.* 15 13–22. 10.22353/mjbs.2017.15.02

[B37] Lei B. Lu K. Ding F. Zhang K. Chen Y. Zhao H. (2014). RNA sequencing analysis reveals transcriptomic variations in tobacco (Nicotiana tabacum) leaves affected by climate, soil, and tillage factors. *Int. J. Mol. Sci.* 15 6137–6160. 10.3390/ijms15046137 24733065 PMC4013620

[B38] Li J. Chory J. (1997). A putative leucine-rich repeat receptor kinase involved in brassinosteroid signal transduction. *Cell* 90 929–938. 10.1016/s0092-8674(00)80357-8 9298904

[B39] Li J. Nagpal P. Vitart V. McMorris T. C. Chory J. (1996). A role for brassinosteroids in light-dependent development of *Arabidopsis*. *Science* 272 398–401. 10.1126/science.272.5260.398 8602526

[B40] Li J. Wen J. Lease K. A. Doke J. T. Tax F. E. Walker J. C. (2002). BAK1, an *Arabidopsis* LRR receptor-like protein kinase, interacts with BRI1 and modulates brassinosteroid signaling. *Cell* 110 213–222. 10.1016/S0092-8674(02)00812-7 12150929

[B41] Liao Y. Smyth G. K. Shi W. (2014). FeatureCounts: an efficient general purpose program for assigning sequence reads to genomic features. *Bioinformatics* 30 923–930. 10.1093/bioinformatics/btt656 24227677

[B42] Lin J. Shao S. Wang Y. Qi M. Lin L. Wang Y. (2016). Germination responses of the halophyte *Chloris virgata* to temperature and reduced water potential caused by salinity, alkalinity and drought stress. *Grass Forage Sci.* 71 507–514. 10.1111/gfs.12218

[B43] Liphschitz N. Adiva-Shomer I. Eshel A. Waisel Y. (1974). Salt glands on leaves of rhodes grass (*Chloris gayana* Kth.). *Ann. Bot.* 38 459–462. 10.1093/oxfordjournals.aob.a084829

[B44] Lu X. Zhou X. Cao Y. Zhou M. McNeil D. Liang S. (2017). RNA-seq analysis of cold and drought responsive transcriptomes of *Zea mays* ssp. Mexicana L. *Front. Plant Sci.* 8:136. 10.3389/fpls.2017.00136 28223998 PMC5293773

[B45] Mobli A. Rinwa A. Sahil Chauhan B. S. (2020). Effects of sorghum residue in presence of preemergence herbicides on emergence and biomass of *Echinochloa colona* and *Chloris virgata*. *PLoS One* 15:e0229817. 10.1371/journal.pone.0229817 32119693 PMC7051094

[B46] Mora-García S. Vert G. Yin Y. Caño-Delgado A. Cheong H. Chory J. (2004). Nuclear protein phosphatases with Kelch-repeat domains modulate the response to brassinosteroids in *Arabidopsis*. *Genes Dev.* 18 448–460. 10.1101/gad.1174204 14977918 PMC359398

[B47] Nakano T. Asami T. (2014). Brassinosteroids signaling and biosynthesis. *Plant Chem. Biol.* 4 128–144. 10.1002/9781118742921

[B48] Neff M. M. Nguyen S. M. Malancharuvil E. J. Fujioka S. Noguchi T. Seto H. (1999). Bas1: a gene regulating brassinosteroid levels and light responsiveness in *Arabidopsis*. *Proc. Natl. Acad. Sci. U.S.A.* 96 15316–15323. 10.1073/pnas.96.26.15316 10611382 PMC24817

[B49] Ngo T. D. Krishnan M. Boutsalis P. Gill G. Preston C. (2018). Target-site mutations conferring resistance to glyphosate in feathertop Rhodes grass (*Chloris virgata*) populations in Australia. *Pest. Manag. Sci.* 47 1094–1100. 10.1002/ps.4512 28019078

[B50] Nguyen L. T. Schmidt H. A. Von Haeseler A. Minh B. Q. (2015). IQ-TREE: a fast and effective stochastic algorithm for estimating maximum-likelihood phylogenies. *Mol. Biol. Evol.* 32 268–274. 10.1093/molbev/msu300 25371430 PMC4271533

[B51] Nishiuchi S. Fujihara K. Liu S. Takano T. (2010). Analysis of expressed sequence tags from a NaHCO3-treated alkali-tolerant plant, *Chloris virgata*. *Plant Physiol. Biochem.* 48 247–255. 10.1016/j.plaphy.2010.01.024 20199868

[B52] Ochoa V. Madrid E. Said M. Rubiales D. Cabrera A. (2015). Molecular and cytogenetic characterization of a common wheat-*Agropyron cristatum* chromosome translocation conferring resistance to leaf rust. *Euphytica* 201 89–95. 10.1007/s10681-014-1190-5

[B53] Ogawa M. Hanada A. Yamauchi Y. Kuwahara A. Kamiya Y. Yamaguchi S. (2003). Gibberellin biosynthesis and response during *Arabidopsis* seed germination. *Plant Cell* 15 1591–1604. 10.1105/tpc.011650 12837949 PMC165403

[B54] Park S. Fung P. Nishimura N. Jensen D. R. Zhao Y. Lumba S. (2009). Abscisic acid inhibits PP2Cs via the PYR/PYL family of ABA- binding START proteins. *Science* 324 1068–1071. 10.1126/science.1173041.Abscisic 19407142 PMC2827199

[B55] Rajjou L. Duval M. Gallardo K. Catusse J. Bally J. Job C. (2012). Seed germination and vigor. *Annu. Rev. Plant Biol.* 63 507–533. 10.1146/annurev-arplant-042811-105550 22136565

[B56] Ramesh K. R. Hemalatha R. Vijayendra C. A. Arshi U. Z. S. Dushyant S. B. Dinesh K. B. (2016). Transcriptome analysis of *Solanum melongena* L. (eggplant) fruit to identify putative allergens and their epitopes. *Gene* 576 64–71. 10.1016/j.gene.2015.09.064 26424595

[B57] Robinson M. D. McCarthy D. J. Smyth G. K. (2009). edgeR: a bioconductor package for differential expression analysis of digital gene expression data. *Bioinformatics* 26 139–140. 10.1093/bioinformatics/btp616 19910308 PMC2796818

[B58] Rodriguez M. C. S. Edsgärd D. Hussain S. S. Alquezar D. Rasmussen M. Gilbert T. (2010). Transcriptomes of the desiccation-tolerant resurrection plant Craterostigma plantagineum. *Plant J.* 63 212–228. 10.1111/j.1365-313X.2010.04243.x 20444235

[B59] Schwartz S. H. Tan B. C. Gage D. A. Zeevaart J. A. D. McCarty D. R. (1997). Specific oxidative cleavage of carotenoids by VP14 of maize. *Science* 276 1872–1874. 10.1126/science.276.5320.1872 9188535

[B60] Sharma H. C. Gill B. S. Uyemoto J. K. (1984). High levels of resistance in *Agropyron* species to barley yellow dwarf and wheat streak mosaic viruses. *J. Phytopathol.* 110 143–147. 10.1111/j.1439-0434.1984.tb03402.x

[B61] Shimada S. Komatsu T. Yamagami A. Nakazawa M. Matsui M. Kawaide H. (2015). Formation and dissociation of the BSS1 protein complex regulates plant development via brassinosteroid signaling. *Plant Cell* 27 375–390. 10.1105/tpc.114.131508 25663622 PMC4456923

[B62] Shimada Y. Fujioka S. Miyauchi N. Kushiro M. Takatsuto S. Nomura T. (2001). Brassinosteroid-6-oxidases from *Arabidopsis* and tomato catalyze multiple C-6 oxidations in brassinosteroid biosynthesis1. *Plant Physiol.* 126 770–779. 10.1104/pp.126.2.770 11402205 PMC111167

[B63] Shin Y.-S. (2013). *Medicinal Plants in Mongolia.* Flora: Wiely, 169–172.

[B64] Sreenivasulu N. Usadel B. Winter A. Radchuk V. Scholz U. Stein N. (2008). Barley grain maturation and germination: metabolic pathway and regulatory network commonalities and differences highlighted by new MapMan/PageMan profiling tools. *Plant Physiol.* 146 1738–1758. 10.1104/pp.107.111781 18281415 PMC2287347

[B65] Stirnberg P. Furner I. J. Ottoline Leyser H. M. (2007). MAX2 participates in an SCF complex which acts locally at the node to suppress shoot branching. *Plant J.* 50 80–94. 10.1111/j.1365-313X.2007.03032.x 17346265

[B66] Stirnberg P. Sande K. Van De Leyser H. M. O. (2002). MAX1 and MAX2 control shoot lateral branching in *Arabidopsis*. *Development* 129 1131–1141. 11874909 10.1242/dev.129.5.1131

[B67] Sun T. P. Kamiya Y. (1994). The *Arabidopsis* GA1 locus encodes the cyclase ent-kaurene synthetase A of gibberellin biosynthesis. *Plant Cell* 6 1509–1518. 10.1105/tpc.6.10.1509 7994182 PMC160538

[B68] Suttie J. M. (2006). *Country Pasture/Forage Resources Profiles: Mongolia.* Rome: FAO.

[B69] Szekeres M. Németh K. Koncz-Kálmán Z. Mathur J. Kauschmann A. Altmann T. (1996). Brassinosteroids rescue the deficiency of CYP90, a cytochrome P450, controlling cell elongation and de-etiolation in *Arabidopsis*. *Cell* 85 171–182. 10.1016/S0092-8674(00)81094-6 8612270

[B70] Talon M. Koornneef M. Zeevaart J. A. D. (1990). Endogenous gibberellins in *Arabidopsis thaliana* and possible steps blocked in the biosynthetic pathways of the semidwarf ga4 and ga5 mutants. *Proc. Natl. Acad. Sci. U.S.A.* 87 7983–7987. 10.1073/pnas.87.20.7983 2236013 PMC54876

[B71] Tang W. Kim T.-W. Oses-Prieto J. A. Sun Y. Deng Z. Zhu S. (2008). Brassinosteroid-Signaling Kinases (BSKs) mediate signal transduction from the receptor kinase BRI1 in *Arabidopsis* NIH Public Access. *Science* 321 557–560. 10.1126/science.1156973 18653891 PMC2730546

[B72] Thomas S. G. Phillips A. L. Hedden P. (1999). Molecular cloning and functional expression of gibberellin 2-oxidases, multifunctional enzymes involved in gibberellin deactivation. *Proc. Natl. Acad. Sci. U.S.A.* 96 4698–4703. 10.1073/pnas.96.8.4698 10200325 PMC16395

[B73] Umehara M. Hanada A. Yoshida S. Akiyama K. Arite T. Takeda-Kamiya N. (2008). Inhibition of shoot branching by new terpenoid plant hormones. *Nature* 455 195–200. 10.1038/nature07272 18690207

[B74] Végh A. Incze N. Fábián A. Huo H. Bradford K. J. Balázs E. (2017). Comprehensive analysis of DWARF14-LIKE2 (DLK2) reveals its functional divergence from 0strigolactone-related paralogs. *Front. Plant Sci.* 8:1641. 10.3389/fpls.2017.01641 28970845 PMC5609103

[B75] Wang Z. Gerstein M. Snyder M. (2009). RNA-Seq: a revolutionary tool for transcriptomics. *Nat. Rev. Genet.* 10 57–63. 10.1038/nrg2484 19015660 PMC2949280

[B76] Wang Z. Y. Seto H. Fujioka S. Yoshida S. Chory J. (2001). BRI1 is a critical component of a plasma-membrane receptor for plant steroids. *Nature* 410 380–383. 10.1038/35066597 11268216

[B77] Wei Y. Xu Y. Lu P. Wang X. Li Z. Cai X. (2017). Salt stress responsiveness of a wild cotton species (*Gossypium klotzschianum*) based on transcriptomic analysis. *PLoS One* 12:e0178313. 10.1371/journal.pone.0178313 28552980 PMC5446155

[B78] Xu W. Li R. Zhang N. Ma F. Jiao Y. Wang Z. (2014). Transcriptome profiling of *Vitis amurensis*, an extremely cold-tolerant Chinese wild *Vitis* species, reveals candidate genes and events that potentially connected to cold stress. *Plant Mol. Biol.* 86 527–541. 10.1007/s11103-014-0245-2 25190283

[B79] Yang C. W. Jianaer A. Li C. Y. Shi D. C. Wang D. L. (2008). Comparison of the effects of salt-stress and alkali-stress on photosynthesis and energy storage of an alkali-resistant halophyte *Chloris virgata*. *Photosynthetica* 46:273. 10.1007/s11099-008-0047-3

[B80] Yang Z. Dai Z. Lu R. Wu B. Tang Q. Xu Y. (2017). Transcriptome analysis of two species of jute in response to polyethylene glycol (PEG)- induced drought stress. *Sci. Rep.* 7 16565–16576. 10.1038/s41598-017-16812-5 29185475 PMC5707433

[B81] Yu F. Wu Y. Xie Q. (2015). Precise protein post-translational modifications modulate ABI5 activity. *Trends Plant Sci.* 20 569–575. 10.1016/j.tplants.2015.05.004 26044742

[B82] Yu Y. Guo G. Lv D. Hu Y. Li J. Li X. (2014). Transcriptome analysis during seed germination of elite Chinese bread wheat cultivar Jimai 20. *BMC Plant Biol.* 14:20. 10.1186/1471-2229-14-20 24410729 PMC3923396

[B83] Yuan J. Li H. Yang Y. (2020). The compensatory tillering in the forage grass hordeum brevisubulatum after simulated grazing of different severity. *Front. Plant Sci.* 11:792. 10.3389/fpls.2020.00792 32595678 PMC7304348

[B84] Zhang J. Liu W. Han H. Song L. Bai L. Gao Z. (2015). De novo transcriptome sequencing of *Agropyron cristatum* to identify available gene resources for the enhancement of wheat. *Genomics* 106 129–136. 10.1016/j.ygeno.2015.04.003 25889708

[B85] Zhao M. Zhang H. Yan H. Qiu L. Baskin C. C. Job D. (2018). Mobilization and role of starch, protein, and fat reserves during seed germination of six wild grassland species. *Front. Plant Sci.* 9:234. 10.3389/fpls.2018.00234 29535748 PMC5835038

[B86] Zhong S. Chai H. Xu Y. Li Y. Ma J. Y. Sun W. (2017). Drought sensitivity of the carbon isotope composition of leaf dark-respired CO2 in C3 (Leymus Chinensis) and C4 (*Chloris virgata* and hemarthria altissima) grasses in northeast China. *Front. Plant Sci.* 8:1996. 10.3389/fpls.2017.01996 29375587 PMC5770615

[B87] Zhou S. Zhang J. Han H. Zhang J. Ma H. Zhang Z. (2019). Full-length transcriptome sequences of *Agropyron cristatum* facilitate the prediction of putative genes for thousand-grain weight in a wheat-*A. cristatum* translocation line. *BMC Genomics* 20:1025. 10.1186/s12864-019-6416-4 31881839 PMC6935218

