# Supplementary material for: Transcriptome Analysis of Chloris virgata, Which Shows the Fastest Germination and Growth in the Major Mongolian Grassland Plant
Source: Front Plant Sci. 2021 Jun 28;12:684987. doi: 10.3389/fpls.2021.684987 (PMC8275185; doi:10.3389/fpls.2021.684987)
Supplement: Supplementary file 1 [file Presentation_1.pdf]

Supplementary Material

1.1 Supplementary Figures

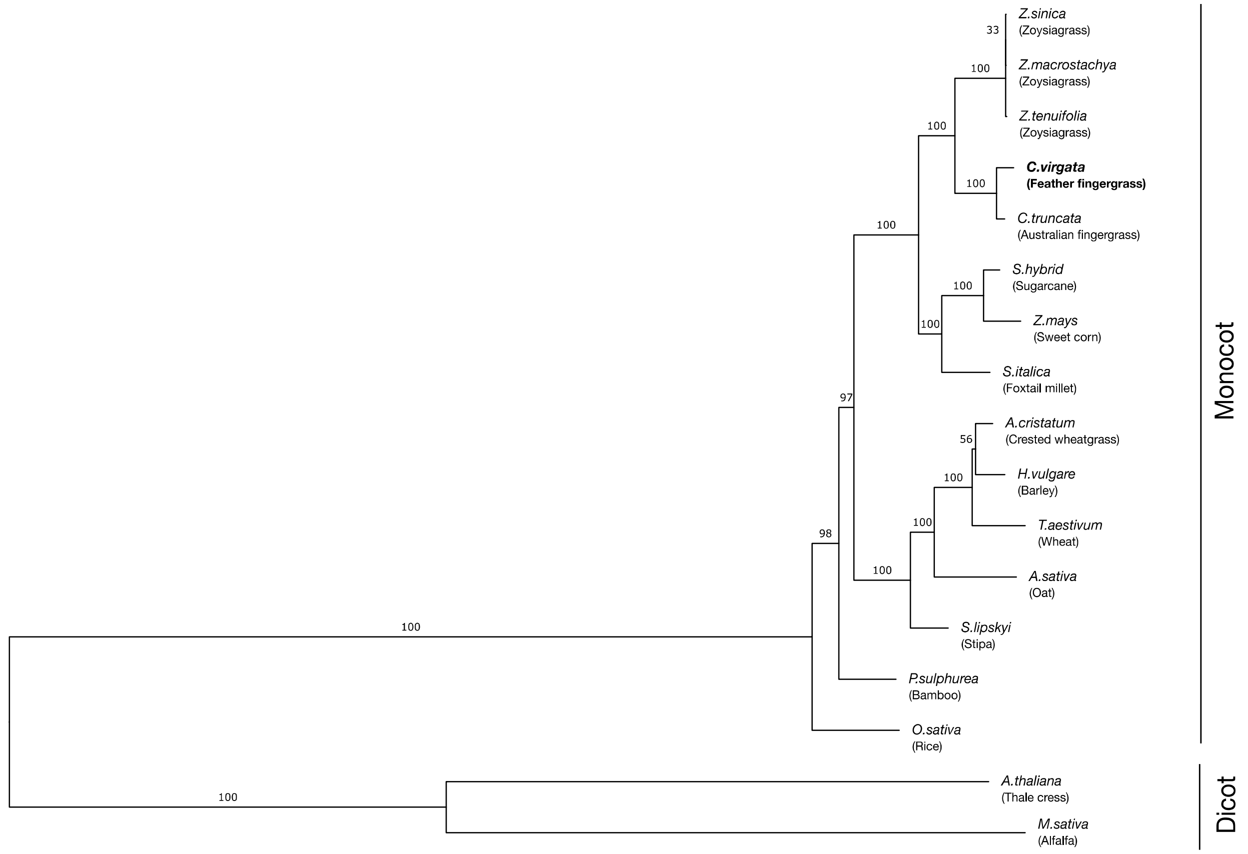

Supplementary Figure 1. Phylogenic analysis of *Chloris virgata* and other common crops using chloroplast genome sequences: The maximum-likelihood phylogenetic tree was constructed using the chloroplast genomes of 17 species including *C. virgata*. Bootstrap values are indicated on the branches. Two dicotyledonous plants, *Arabidopsis thaliana* and *Medicago sativa*, were used as the outgroup. Each plant is indicated with the abbreviated scientific name and common English name. Abbreviations of scientific names and accession numbers on RerSeq and Genbank are as follows: *Zoysia sinica*: NC_042187.1, *Zoysia macrostachya*: NC_042189.1, *Zoysia tenuifolia*: NC_042188.1, *Chloris virgata*: NC_032034.1, *Chloris truncata*: NC_032033.1, *Saccharum hybrid*: NC_029221.1, *Zea mays*: NC_001666.2, *Setaria italica*: MK348609.1, *Agropyron cristatum*: MN703668.1, *Hordeum vulgare*: KT962228.1, *Triticum aestivum*: NC_002762.1, *Avena sativa*: MG687313.1, *Stipa lipskyi*: NC_028444.1, *Phyllostachys sulphurea*: NC_024669.1, *Oryza sativa*: NC_031333.1, *Arabidopsis thaliana*: NC_000932.1, *Medicago sativa*: KU321683.1.

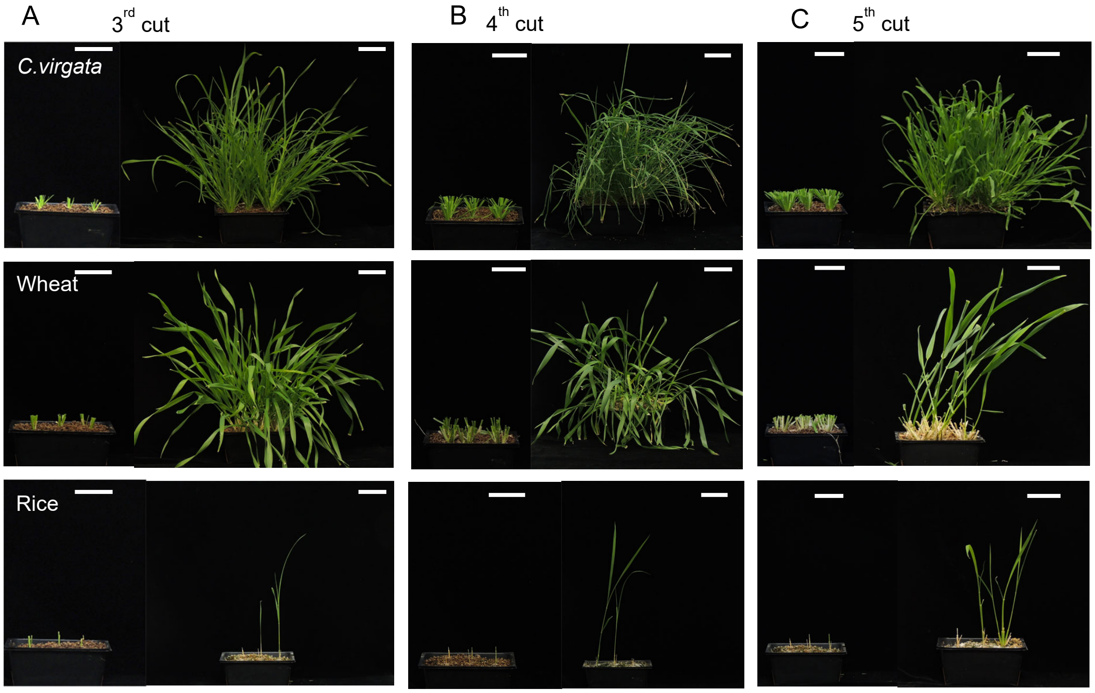

Supplementary Figure 2. *Chloris virgata* possessed higher regeneration ability after shoot cutting than the common crops, wheat and rice. (A) *C. virgata*, rice, and wheat shoots, regrown after the second cutting, were recut a third time (left). The plants were regrown for 2 weeks after the third cutting treatment (right). **(B)** Then, the shoots were recut again (left) for the fourth time. The plants were regrown for 2 weeks after the fourth cutting treatment (right). **(C)** The regrown shoots after the fourth cutting treatment were recut for a fifth time (left). The plants were regrown for 2 weeks (right) after the fifth cutting treatment. Scale bars: 5 cm.

1.2 Supplementary Data Sheest 1 to 4

Supplementary Data 1. All gene ontology terms of differentially expressed genes enriched in the germination stage (2 days after germination), early young developing stage (8 days after germination), young developing stage (17 days after germination), and adult developing stage (28 days after germination) of *C. virgata*. Gene ontology terms were selected from the biological process categories. A false discovery rate (FDR) of 0.03 was used.

Supplement TABLE S1. The expression level of Gibberellin signaling and biosynthesis homologous genes in each development stages of *Chloris virgata*. Possible signaling and biosynthesis genes of gibberellin were identified and expression levels were calculated in each development stages of *C. virgata.*

Gibberellin signaling genes					
Gene name	*A.thaliana*	*O.sativa*	*C.virgata*	Ratio of expression level	
				2d/8d	28d/17d
GID1A<br>GID1B	At3g05120<br>At3g63010	Os05g0407500	Chloris4812c000010	0.569	0.910
SLY	At4g24210	Os02g0580300	Chloris1845c000010	1.110	1.295
GAI<br>RGA<br>RGL1	At1g14920<br>At2g01570<br>At1g66350	Os03g0707600<br>–<br>Os03g0707600	Chloris3604c000010	1.501	0.678
RGL2<br>RGL3	At3g03450<br>At5g17490	Os03g0707600	Chloris25649c000010	1.046	0.990
Gibberellin biosynthesis genes					
Gene name	*A.thaliana*	*O.sativa*	*C.virgata*	Ratio of expression level	
				2d/8d	28d/17d
CPS	At4g02780	Os02g0278700	Chloris5831c000010	5.512	0.907
KS	At1g79460	Os04g0611800	Chloris1808c000010	1.087	1.077
CYP701A3	At5g25900	Os06g0569900	Chloris20633c000010	0.852	1.091
CYP88A	At1g05160	Os06g0110000	Chloris29327c000010	0.709	1.230
			Chloris7975c000010	0.627	0.575
GA20-ox2	At5g51810	Os01g0883800	Chloris27199c000010	0.783	0.992
GA3-ox1	At1g15550	Os05g0178100	Chloris7778c000010	1.720	1.520
GA2-ox1	At1g78440	Os05g0158600	Chloris20434c000010	0.383	0.406
GA2-ox8	At4g21200	-	Chloris26078c000010	1.172	1.683

Supplement TABLE S2. The expression level of Abscisic acid signaling and biosynthesis homologous genes in each development stages of *Chloris virgata*. Possible signaling and biosynthesis genes of abscisic acid were identified and expression levels were calculated in each development stages of *C. virgata.*

ABA signaling genes					
Gene name	*C.virgata*	Expression level			
		2d	8d	17d	28d
PYL1	Chloris6771c000010	12.318	14.309	9.142	10.725
PYL2	Chloris15678c000010	7.134	3.230	3.913	4.008
PYL5	Chloris15680c000010	18.753	12.252	29.315	11.542
SnRK2B	Chloris13011c000010	8.188	15.350	13.383	13.962
SnRK2E	Chloris2715c000010	15.435	16.081	17.145	20.972
ABI1	Chloris27073c000010	1.960	2.740	2.785	2.746
ABI4	Chloris28360c000010	7.200	2.266	3.294	2.330
ABI5	Chloris7667c000010	2.722	4.149	3.415	3.847
ABA biosynthesis genes					
Gene name	*C.virgata*	Expression level			
		2d	8d	17d	28d
NCED1	Chloris2871c000010	42.252	184.852	92.407	128.397
NCED2	Chloris28063c000010	6.649	118.565	115.584	186.908
NCED5	Chloris11441c000010	1.711	2.429	2.197	2.112
NCED6	Chloris2871c000010	42.252	184.852	92.407	128.397
NCED9	Chloris28516c000010	3.287	2.809	2.440	2.371
ABA2	Chloris6223c000010	3.836	3.937	4.964	6.191
AAO2<br>AAO3<br>AAO4	Chloris17143c000010	18.701	17.412	31.502	21.347
CYP707A	Chloris18677c000010	5.905	7.968	9.210	10.682

Supplement TABLE S3. The expression level of Brassinosteroid signaling and biosynthesis homologous genes in each development stages of *Chloris virgata*. Possible signaling and biosynthesis genes of brassinosteroid were identified and expression levels were calculated in each development stages of *C. virgata.*

Brassinosteroid signaling genes					
Gene name	*C.virgata*	Expression level			
		2d	8d	17d	28d
BRI1	Chloris3177c000010	10.956	17.810	15.021	10.993
BRL1	Chloris17044c000010	0.535	0.299	0.366	0.368
BAK1	Chloris23503c000010	99.884	188.794	147.833	224.452
BKI1	Chloris17074c000010	2.859	3.320	3.626	3.495
BSK1	Chloris3139c000010	3.744	5.770	6.783	6.838
BSU1	Chloris13780c000010	23.973	41.229	40.916	47.346
BIN2	Chloris6135c000010	5.152	4.515	5.303	5.825
BSS1	Chloris13348c000010	12.227	8.607	12.169	12.898
BIL1/BZR1 BES1	Chloris30620c000010	9.342	6.826	7.659	10.052
Brassinosteroid biosynthesis genes					
Gene name	*C.virgata*	Expression level			
		2d	8d	17d	28d
DET2	Chloris12539c000010	1.001	1.564	1.808	2.164
DWF4	Chloris405c000010	5.176	6.076	15.432	5.700
CPD	Chloris6902c000010	15.486	19.062	18.119	18.107
BR6ox	Chloris14424c000010	4.920	5.215	3.879	5.854
BAS1	Chloris30576c000010	53.205	16.519	23.069	35.583

Supplement TABLE S4. The expression level of Strigolactone signaling and biosynthesis homologous genes in each development stages of *Chloris virgata*. Possible signaling and biosynthesis genes of strigolactone were identified and expression levels were calculated in each development stages of *C. virgata.*

Strigolactones signaling genes					
Gene name	*C.virgata*	Expression level			
		2d	8d	17d	28d
D14	Chloris9047c000010	16.708	89.922	102.650	63.061
DLK2	Chloris3102c000010	1.766	2.132	2.356	1.869
	Chloris16862c000010	1.705	1.138	6.757	2.429
	Chloris16863c000010	0.988	1.368	4.271	1.689
MAX2	Chloris21103c000010	11.221	8.619	11.184	11.351
SKP1	Chloris20528c000010	137.266	184.025	158.868	192.362
BRC1	Chloris9240c000010	10.025	25.485	18.234	19.146
SMXL7	Chloris14785c000010	16.684	44.417	60.627	97.504
Strigolactones biosynthesis genes					
Gene name	*C.virgata*	Expression level			
		2d	8d	17d	28d
D27	Chloris8648c000010	5.430	5.688	5.052	6.336
MAX3	Chloris8839c000010	2.271	1.756	1.604	2.097
MAX4	Chloris11425c000010	1.130	1.096	1.884	3.721
	Chloris20143c000010	0.513	0.944	0.669	1.537
MAX1	Chloris23859c000010	0.502	0.542	0.575	0.458
CYP711A2<br>CYP711A3	Chloris16015c000010	4.512	4.280	4.016	5.991
